# Umbrella review of photodynamic therapy for cancer: efficacy, safety, and clinical applications

**DOI:** 10.3389/fonc.2025.1528314

**Published:** 2025-08-04

**Authors:** Hanhan Chen, Honglin Li, Hui-Jie Li, Zhen Zhang

**Affiliations:** ^1^ Department of Breast Surgery, Affiliated Hospital of Shandong University of Traditional Chinese Medicine, Jinan, Shandong, China; ^2^ Traditional Chinese Medicine, Shandong Provincial Hospital Affiliated to Shandong First Medical University, Jinan, Shandong, China; ^3^ Department of Oncology, Affiliated Hospital of Shandong University of Traditional Chinese Medicine, Jinan, Shandong, China; ^4^ Department of Radiation Oncology, The Third Affiliated Hospital of Shandong First Medical University Affiliated Hospital of Shandong Academy of Medical Sciences, Jinan, Shandong, China

**Keywords:** photodynamic therapy, cancer, meta-analysis, umbrella review, photosensitizer

## Abstract

**Background:**

Photodynamic therapy (PDT) can target cancers, while causing little damage to surrounding healthy tissues

**Objective:**

To systematically evaluate the efficacy, safety, and clinical applications of PDT across cancer types.

**Methods:**

PubMed, EMBASE, Cochrane Library, and Web of Science were searched to April 7, 2024 for systematic reviews and meta-analyses of PDT in patients with cancer. Quality assessment was performed using Assessment of Multiple Systematic Reviews 2, overlapping meta-analyses were handled using Corrected Covered Area, and data re-synthesized using a random-effects model.

**Results:**

Eighteen publications met the inclusion criteria. There is weak evidence that PDT combined with biliary stenting improves overall survival (OS) relative to stenting alone (hazard ratio (HR) 0.49, 95% confidence interval (CI) 0.33–0.73), while PDT with chemotherapy improved OS (HR 0.36, 95% CI 0.15–0.87), without increasing adverse events. Weak evidence indicated lower clearance and complete response rates and higher recurrence rates of non-melanoma skin cancers, particularly basal cell carcinoma (BCC), after PDT than following surgery. In squamous cell carcinoma (SCC), complete response rates (relative risk 2.75; 95% CI 2.19–3.45) were higher for laser-assisted than conventional PDT; PDT provided better cosmetic outcomes than other therapies. Single-arm meta-analyses demonstrated some efficacy of PDT for treating cutaneous metastatic SCC, oral SCC, prostate cancer, and bladder cancer.

**Conclusion:**

PDT shows potential benefits in several cancers, especially for non-melanoma skin cancer and unresectable cholangiocarcinoma. While newer PDT strategies may improve outcomes, more high-quality trials are needed to confirm its role across cancer types.

**Systematic review registration:**

https://www.crd.york.ac.uk/PROSPERO/view/CRD42024538243, identifier CRD42024538243.

## 1 Introduction

Cancer remains a global threat to human health due to its complex biological characteristics and effects in increasing morbidity and mortality (1). Although remarkable progress has been made in surgical techniques, chemotherapy, radiation therapy, and immunotherapy, which have significantly extended the survival times of cancer patients, there remains an urgent need for treatments that are both safer and more effective (2). Photodynamic therapy (PDT) has garnered significant attention as a promising cancer treatment (3), as it offers localized effects with minimal damage to surrounding healthy tissue, achieved using photosensitizers that generate reactive oxygen species (ROS) able to induce cell death under specific light wavelengths (4). PDT involves synergistic effects of photochemistry and photobiology, and offers an alternative to traditional cancer treatments (5).

PDT has transitioned from experimental studies to active clinical investigation, with over 60 registered clinical trials (**Supplementary Table S1**) currently evaluating its efficacy across various cancer types in the last decade (6–8). Compared with conventional cancer treatments, PDT offers several distinct advantages. PDT employs noninvasive or minimally invasive techniques that effectively minimize collateral damage to healthy tissues. Moreover, PDT is associated with mild side effects, and can be repeatedly administered based on the patient’s clinical condition. Notably, PDT can be synergistically combined with other cancer treatment methods to enhance overall therapeutic efficacy while preserving the inherent benefits of the adjunct treatments. With ongoing advances in treatment protocols and innovative photosensitizer delivery technologies, PDT as a minimally invasive and precisely targeted treatment approach—holds significant potential to advance cancer therapy, improve patients’ quality of life, and increase the likelihood of recovery.

The therapeutic potential of PDT has been explored in various cancers, including skin, lung, esophageal, and head and neck tumors (9). The multiple mechanisms underlying PDT, including direct tumor cell killing, disruption of tumor blood vessels, and activation of immune responses, underscore its versatility (10). Despite promising results in many studies, the clinical application of PDT has been inconsistent, possibly due to variability in treatment regimens and differences attributable to cancer types and stages (8). Therefore, there is a pressing need to synthesize the existing research evidence to comprehensively evaluate the overall efficacy, safety, and clinical applicability of PDT across different cancer types.

Umbrella reviews of systematic reviews and meta-analyses can address this need by providing a high-level overview of current evidence, through integration and critical analysis of existing research data (11). In this study, we systematically evaluated the evidence from previous systematic reviews and meta - analyses to review the application of PDT in cancer treatment. Through this thorough analysis, our aim was to clarify the potential of PDT in cancer treatment and to lay a solid foundation for future research and clinical practice.

## 2 Materials and methods

This study was conducted in accordance with the PRISMA guidelines (12) and its protocol was registered with PROSPERO (registration number: CRD42024538243).

### 2.1 Search strategy

A comprehensive search was performed across multiple electronic databases, including PubMed, EMBASE, Cochrane Library, and Web of Science, up to April 7, 2024. Additionally, reference lists of eligible studies were scrutinized to identify supplementary sources. The primary search terms employed were “photodynamic therapy,” “cancer,” “meta-analysis,” and “systematic review.” The specific search strategies and corresponding results for PubMed are detailed in **Supplementary Table S2**.

### 2.2 Inclusion and exclusion criteria

For the selection of studies, the following inclusion criteria were applied: (1) The study design was a systematic review or meta-analysis, with either a single-arm or two-arm; (2) Participants were individuals diagnosed with cancer; (3) The intervention under investigation was PDT; (4) The control group received treatments such as surgery, cryotherapy, chemotherapy, placebo, or PDT with alternative photosensitizers; (5) The study reported at least one outcome measure related to efficacy or safety, including overall survival (OS), recurrence rate, response rate, or adverse events (AEs).

Conversely, studies were excluded based on the following criteria: (1) Animal or *in vitro* experiments; (2) Case reports; (3) Abstracts without full-text availability; (4) Original clinical trials; (5) Systematic reviews lacking a meta-analysis component; (6) Network meta-analyses; (7) Studies focused on cancer prevention; (8) Studies involving patients with precancerous lesions; (9) Meta-analyses where the forest plots did not provide data from individual studies.

### 2.3 Literature screening and data extraction

The literature screening process was performed independently by two reviewers (HHC and HLL) in accordance with the established inclusion and exclusion criteria. The initial phase of screening involved evaluating the titles and abstracts of identified publications. Subsequently, eligible studies were selected through a full-text review. All excluded studies and the corresponding reasons for their exclusion were meticulously recorded. In instances where discrepancies arose between the two reviewers, a third reviewer (ZZ) was consulted to facilitate the resolution of disagreements and achieve consensus.

The following data were extracted from the selected studies: first author, publication year, number of included studies and patients, cancer type, treatment modality, photosensitizers used, outcome measures, combined effect size with corresponding 95% confidence interval (CI) values, heterogeneity metrics, P-values, funding sources, and quality assessment tools. Additionally, for each original study included in the forest plots, data on the authors, publication year, treatment, sample size, outcome measures, analysis models, and effect size with 95% CI values were extracted. If such information was not available in the forest plots, it was obtained by tracing back to the original clinical studies.

### 2.4 Quality assessment

The methodological quality of the included studies was independently evaluated by two reviewers (HHC and HLL) using the Assessment of Multiple Systematic Reviews (AMSTAR) 2 tool, which comprises 16 items (13). Among these items, domains 2, 4, 7, 9, 11, 13, and 15 are designated as critical. Studies with no or only one non-critical weakness were classified as high quality. Those with multiple non-critical weaknesses were assigned a moderate quality rating. Studies containing one critical flaw, regardless of the presence of non-critical weaknesses, were rated as low quality. Conversely, studies with multiple critical flaws were considered very low quality, irrespective of any non-critical weaknesses. Any discrepancies between the reviewers were resolved through discussion with a third reviewer (ZZ) to achieve consensus.

### 2.5 Removal of overlapping meta-analyses

The increasing number of meta-analyses has resulted in a proliferation of analyses addressing the same research questions, leading to overlapping primary studies and the potential for bias. To mitigate this issue, an established approach was employed to quantify the degree of overlap between studies using citation matrices and the Corrected Covered Area (CCA) (14, 15). Specifically, when the CCA exceeded 15%, the publication with the larger number of included studies and higher methodological quality was selected for inclusion. Conversely, when the CCA was less than 15%, both overlapping publications were retained.

### 2.6 Classification of evidence

The evidence classification system was established according to methodological precedents (16), employing a tiered categorization framework:

Class I (convincing evidence) featured a highly significant pooled effect size (*P* < 10^−6^), a significant effect in the largest study (*P* < 0.05), low heterogeneity (I^2^ < 50%), a 95% prediction interval (PI) excluding the null value, no significant publication bias (*P* > 0.1) as indicated by Egger’s test, and included over 1,000 patients in the meta-analysis.

Class II (highly suggestive evidence) included a highly significant pooled effect size (p < 10^−6^), a significant effect in the largest study (*P* < 0.05), and more than 1,000 patients in the meta-analysis. And did not meet at least one of the following conditions: low heterogeneity (I^2^ < 50%), a 95% prediction interval (PI) excluding the null value, and no significant publication bias (*P* > 0.1) as indicated by Egger’s test.

Class III (suggestive evidence) was characterized by a significant pooled effect size (*P* < 10^−3^), more than 1,000 patients in the meta-analysis, and no significant effect in the largest study (*P* > 0.05). And did not meet at least one of the following conditions: low heterogeneity (I^2^ < 50%), a 95% prediction interval (PI) excluding the null value, no significant publication bias (*P* > 0.1) as indicated by Egger’s test.

Class IV (weak evidence) showed a significant pooled effect size (*P* < 0.05), and less than 1,000 patients in the meta-analysis. And did not meet at least one of the following conditions: low heterogeneity (I^2^ < 50%), a 95% prediction interval (PI) excluding the null value, no significant publication bias (*P* > 0.1) as indicated by Egger’s test, a significant effect in the largest study (*P* < 0.05).

ns (non-significant evidence) lacked a significant pooled effect size (*P* > 0.05).

### 2.7 Statistical analysis

The selection of analysis methods was determined by the number of studies included in each meta-analysis. Specifically, for meta-analyses comprising five or more studies, the DerSimonian-Laird (DL) method was utilized (17). Conversely, for meta-analyses with fewer than five studies, the Hartung-Knapp-Sidik-Jonkman method was employed. This preference was based on the tendency of the DL method to underestimate the 95% CI when the number of studies is limited (18, 19). Heterogeneity among studies was assessed using the I² statistic, with values exceeding 50% indicating significant heterogeneity. Additionally, 95% PI were calculated to estimate the range of true effects that might be expected in future studies.

Publication bias was evaluated using Egger’s regression test and contour-enhanced funnel plots. A *P*-value of less than 0.1 from Egger’s test was considered indicative of potential small-study effects. In cases where publication bias was detected, the “trim-and-fill” method was applied to adjust the effect size and 95% CI values. Furthermore, the test of excess significance was conducted to determine whether the number of significant findings exceeded the number expected by chance, with *P* < 0.1 suggesting potential bias.

All statistical analyses were performed using R software (version 4.3.3) and the “metaumbrella” package (20, 21), with two-tailed *P*-values used to determine statistical significance.

## 3 Results

### 3.1 Literature selection

**Figure 1:**
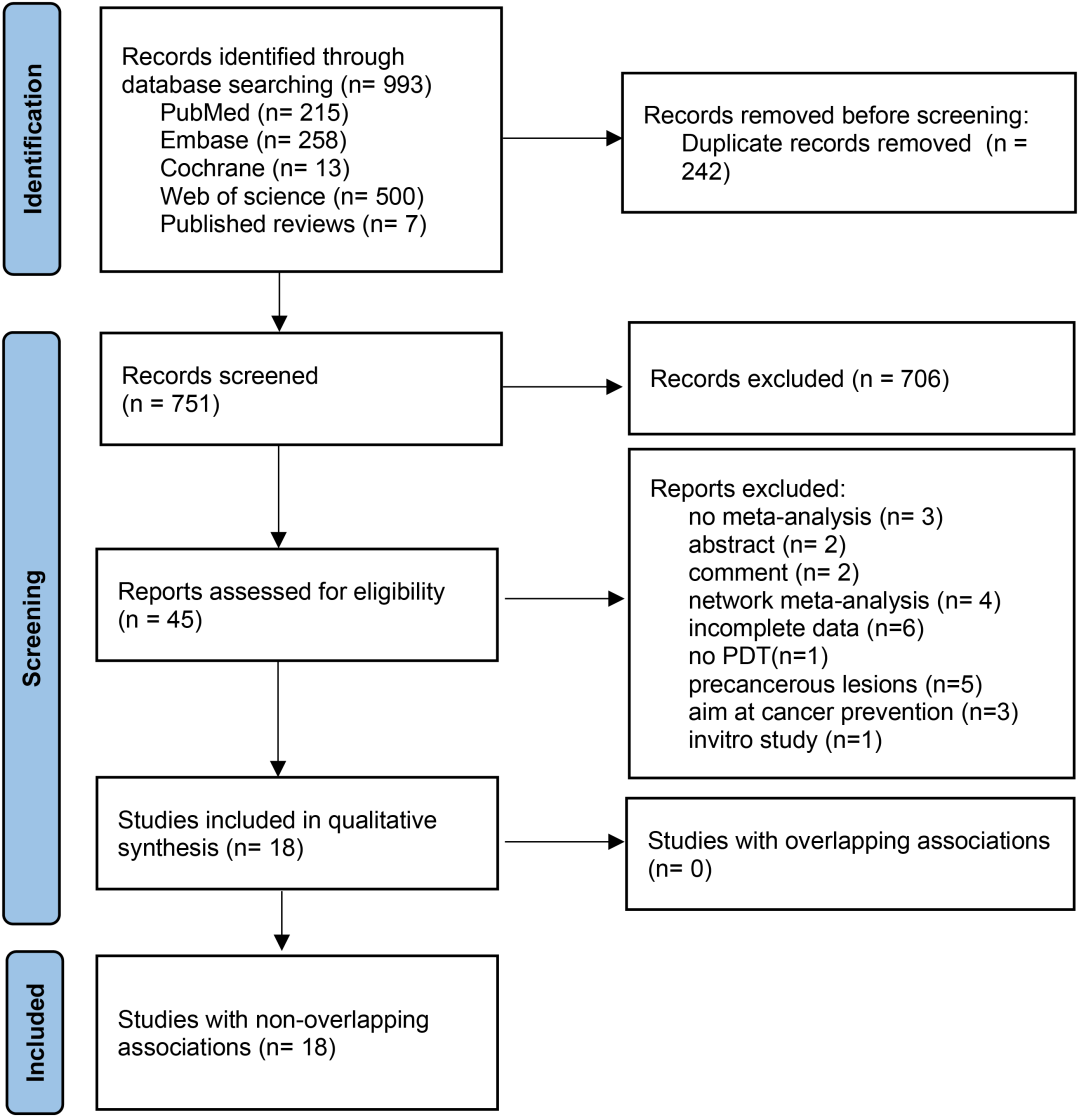
Flow chart for the screening of publications. PDT, photodynamic therapy.

**Figure 1** shows the literature screening process. From 993 identified publications, 242 duplicates were removed. After title and abstract screening, 706 publications were excluded. Full-text reviews identified 18 eligible publications. Details on excluded studies and reasons for exclusion are provided in **Supplementary Table S3**.

### 3.2 Basic characteristics of included studies

A comprehensive summary of the 18 included publications (22–39) is presented in **Table 1**. First authors were from the UK (n = 2), the USA (n = 2), and China (n = 14). Cancers studied include cholangiocarcinoma (n = 4), skin cancer (n = 8), prostate cancer (n = 2), bladder cancer (n = 2), oral cancer (n = 1), and cutaneous metastases (n = 1). The number of primary studies included in these publications ranged from 5 to 28, with sample sizes between 40 and 2327. Reported outcome measures included OS, complete response, recurrence rate, mortality, Karnofsky performance status, cosmetic outcome, and AEs. The photosensitizers and parameters used for different cancers are shown in **Supplementary Table S4**.

**Table 1:** Basic characteristics of included publications.

Author (year)	Country	Cancer type	Photosensitizer	Study design	Intervention	Comparison	No. of included studies	No. of patients	Outcome	Metric	Funding	Quality appraisal tool	Reporting guidelines	AMSTA 2
Leggett (2012) (22)	USA	Unresectable cholangiocarcinoma	Porphyrin derivative	RCT; Cohort	Biliary stenting with PDT	Biliary stenting	6	327	Length of survival; Mortality; KPS; Serum bilirubin	RR; WMD	NA	NA	NA	CL
Lansbury (2013) (23)	UK	Non-metastatic squamous cell carcinoma of the skin	ALA; MAL; Mthpc; Hematoporphyrin derivative	Non-comparative studies	PDT	NA	14	273	Complete response; Recurrence	Rate	NA	Joerg Albrecht for reporting case series and case reports	MOOSE	H
Spratt (2014) (24)	USA	Cutaneous metastases from advanced cancer (breast, adenocarcinoma)	NR	RCT; Cohort; Case series	PDT	NA	5	40	Complete response; Objective response; Recurrence rate	Rate	NA	Jadad	NR	L
Lu (2015) (25)	China	Unresectable cholangiocarcinoma	Photofrin; Photosan-3	RCT; Cohort	PDT with stenting	Stent alone	8	642	OS	RR	NA	NA	NR	CL
Wang (2015) (26)	China	BCC	ALA; MAL	RCT	PDT	Surgery; Cryotherapy; Topical therapy; Placebo	8	1583	Complete clearance rate; Recurrence rate; Cosmetic outcome	RR	NA	Cochrane risk of bias tool	NR	CL
Zou (2016) (27)	China	BCC	ALA; MAL	RCT	PDT	Surgical excision	5	596	Complete response rate; Probability of recurrence	RR	NA	Jadad	PRISMA	L
Collier (2018) (28)	UK	BCC	ALA; MAL	RCT	PDT	Surgery; Cryotherapy; Topical therapy; Placebo	19	2327	Cosmetic outcome; 3-month initial clearance	RR	NA	Cochrane risk of bias tool	PRISMA	L
Wang (2019) (29)	China	Prostate cancer	Temoporfin; 5-ALA; Motexafin lutetium; Temoporfin; Padoporfin; Padeliporfin	RCT; Single arm	PDT	NR	14	654	Biopsy-negative rate; PSA decreasing rate	Rate	NA	Agency for Healthcare Research and Quality score	NA	CL
Gu (2021) (30)	China	Non-melanoma skin cancers	NR	RCT	Laser-assisted PDT	Conventional PDT	5	267	Complete response rate	RR	NA	Jadad	PRISMA	L
Wang (2020) (31)	China	BCC	NR	RCT; Retrospective	MAL-PDT	Surgery; ALA-PDT; Placebo; Cryotherapy; Imiquimod	8	1339	Complete response; Recurrence; Cosmetic outcome	RR	Y	Cochrane risk of bias tool	PRISMA	CL
Zhong (2020) (32)	China	Bowen’s disease	NR	RCT	PDT	5-FU; Cryotherapy	12	446	Efficacy (lesion reduction); Recurrence rate	RR	NA	Cochrane risk of bias tool	MOOSE	L
Guo (2021) (33)	China	Prostate cancer	NR	Single arm	Vascular-targeted PDT	NA	7	733	Positive biopsy; Biochemical recurrence-free survival	Rate	NA	NR	PRISMA; MOOSE	L
Lin (2021) (34)	China	Oral squamous cell carcinoma	NR	Single arm	PDT	Talaporfin sodium; Porfimer sodium; HPPH; mTHPC; HPD	18	900	Complete response; Recurrence rate; Overall response	Rate	Y	Downs–Black checklist	PRISMA	L
Chen (2022) (35)	China	Hilar cholangiocarcinoma	NR	Cohort	PDT with stenting	Stenting alone	6	446	Survival rate; OS; Adverse events	Rate; HR; OR	NA	Risk of bias in non-randomized Studies of interventions tool	PRISMA	CL
Li (2023) (36)	China	Non-muscle-invasive bladder cancer	Photosens; ALA; TLD-1433; Radachlorin; Ce6PVP; Photogeme	Single arm	PDT	NA	28	648	Complete response; Recurrence-free rate	Rate	Y	Joanna Briggs Institute	PRISMA	CL
Ou-yang (2023) (37)	China	Skin carcinomas	ALA; MAL	RCT	ALA-PDT; MAL-PDT	ALA-PDT; MAL-PDT;5-FU; Imiquimod; Cryotherapy; Surgery; Placebo; YAG-AFL-PDT;	21	2166	Response; Recurrence; Cosmetic rate; Adverse events; Pain	RR	NA	Cochrane risk of bias Tool	PRISMA	L
Yu (2023) (38)	China	Unresectable extrahepatic cholangiocarcinoma	Photosan; Photofrin; Foscan	RCT; RCS	PDT with chemotherapy	PDT alone or chemotherapy alone	7	542	OS; Adverse events	HR; OR	Y	Cochrane risk of bias tool; NOS	PRISMA	L
Xue (2022) (39)	China	Bowen’s Disease	ALA; MAL	RCT	PDT	5-FU; Cryotherapy; Placebo	8	412	Complete response rate	RR	NA	Cochrane risk of bias tool	PRISMA	CL

ALA, 5-Aminolevulinic acid; AMSTAR 2, assessment of multiple systematic reviews; BCC, basal cell carcinoma; CL, critical low; CI, confidence interval; DL, DerSimonian-Laird; HR, hazard ratio; KPS, Karnofsky performance scale; L, low; MAL, methyl aminolevulinate; NA, not available; NR, not reported; OR, odds ratio; OS, overall survival; PDT, photodynamic therapy; RCT, randomized controlled trial; RCS, retrospective cohort study; RR, risk ratio; WMD, weighted mean difference; Y, yes; YAG-AFL, erbium: yttrium-aluminum-garnet ablative factional laser; 5-FU, 5-Fluorouracil.

### 3.3 Quality assessment

**Supplementary Figure S1** presents methodological quality assessment of the 18 publications. Among them, 1 publication was rated as high quality, 9 as low quality, and 8 as very low quality. Five publications (27.8%) did not predefine a study protocol, 16 (88.9%) did not provide a list of excluded studies with reasons, 1 (5.6%) did not use appropriate tools to assess the risk of bias for each included study, and 4 (22.2%) did not investigate or test for publication bias.

### 3.4 Evidence related to PDT from paired meta-analyses

#### 3.4.1 Cholangiocarcinoma

**Figure 2:**
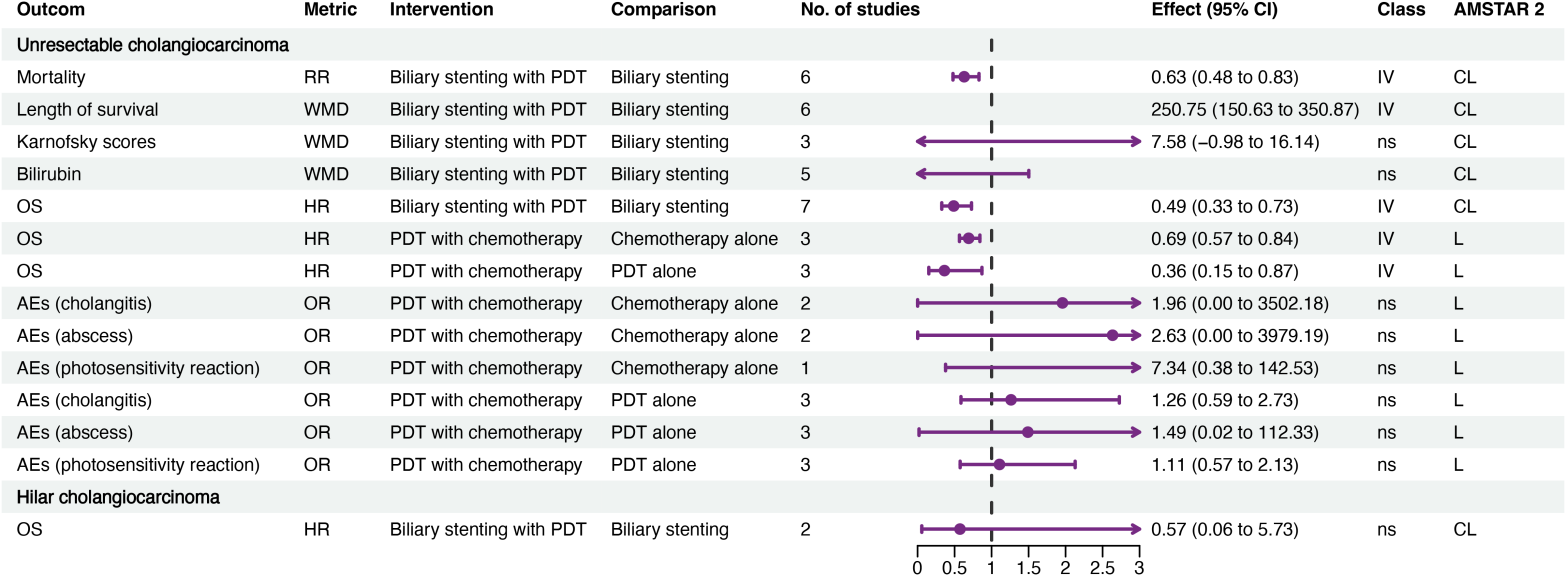
Forest plot of efficacy and AEs of PDT for cholangiocarcinoma. AEs, adverse events; CL, critical low; CI, confidence interval; HR, hazard ratio; L, low; OR, odds ratio; OS, overall survival; PDT, photodynamic therapy; RR, risk ratio; IV, weak evidence (class IV); ns, non-significant (class ns).

Fourteen associations related to unresectable cholangiocarcinoma and one to hilar cholangiocarcinoma were identified (**Figure 2**; **Supplementary Table S4**). The data provided weak evidence that combined PDT with biliary stenting may enhance OS compared to biliary stenting alone (hazard ratio (HR) 0.49, 95% CI 0.33–0.73), potentially extending survival by approximately 250 days. Additionally, there was weak evidence that combining PDT with chemotherapy may further improve OS of patients with cholangiocarcinoma compared to either treatment alone; however, PDT did not result in an improvement in OS for patients with hilar cholangiocarcinoma. Further, combination PDT and chemotherapy did not increase the risk of AEs such as cholangitis, abscess, or photosensitivity reactions.

#### 3.4.2 Non-melanoma skin cancer

We identified 49 associations related to skin cancer, categorized by outcome and cancer type, including basal cell carcinoma (BCC), squamous cell carcinoma (SCC), and Bowen’s disease.

**Figure 3:**
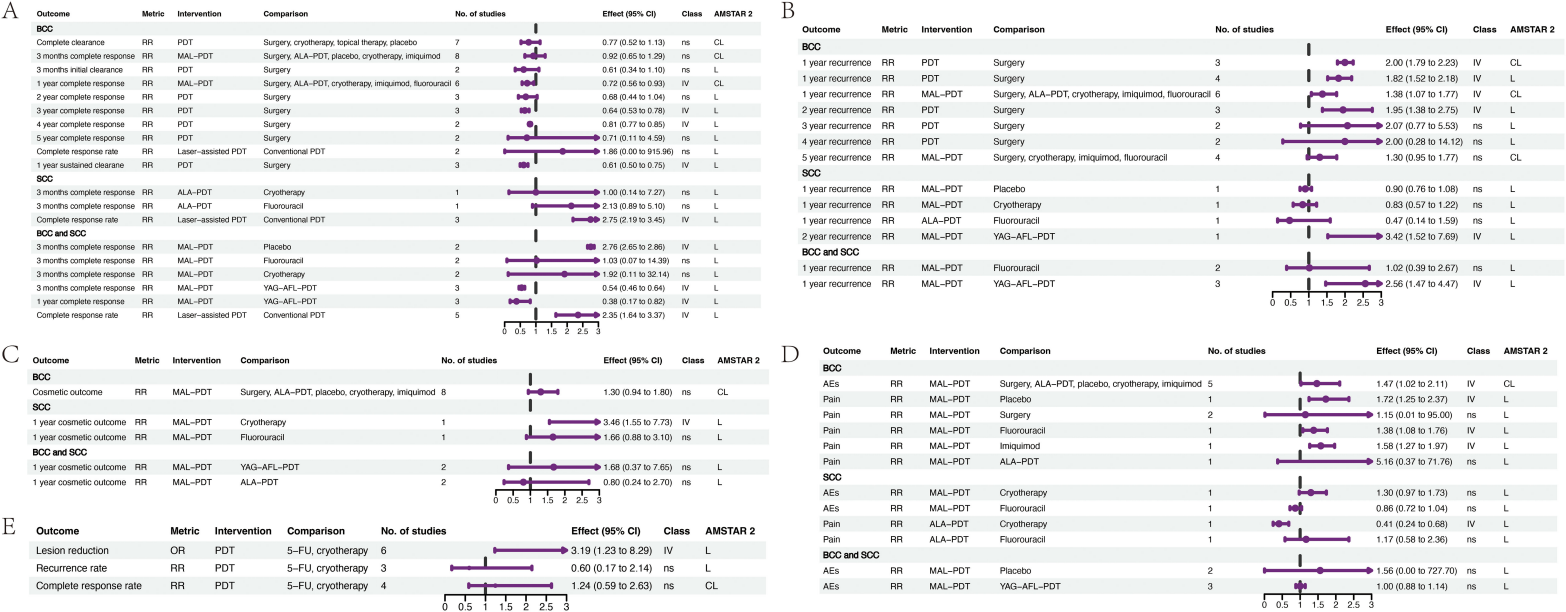
Forest plots of the efficacy of PDT for skin cancer. **(A)** the efficacy of PDT for BCC and SCC. **(B)** the recurrence rate of PDT for BCC and SCC. **(C)** the cosmetic outcome of PDT for BCC and SCC. **(D)** the AEs of PDT for BCC and SCC. **(E)** Bowen’s disease. ALA, 5-Aminolevulinic acid; BCC, basal cell carcinoma; SCC, squamous cell carcinoma; CL, critical low; CI, confidence interval; L, low; MAL, methyl aminolevulinate; OR, odds ratio; PDT, photodynamic therapy; RR, risk ratio; YAG-AFL, erbium: yttrium-aluminum-garnet ablative factional laser; IV, weak evidence (class IV); ns, non-significant (class ns); 5-FU, 5-Fluorouracil.

Nineteen associations related to efficacy were identified (**Figure 3A**; **Supplementary Table S5**), with outcomes including complete clearance rate, complete response rate, and sustained clearance rate. For BCC, weak evidence suggested that PDT results in a lower sustained clearance rate at 1-year (relative risk (RR) 0.61, 95% CI 0.50–0.75), and lower complete response rates at 3-years (RR 0.64, 95% CI 0.53–0.78) and 4-years (RR 0.81, 95% CI 0.77–0.85), than surgery. Additionally, the 1-year complete response rate for methyl aminolevulinate (MAL)-PDT was lower than that for other therapies (RR 0.72, 95% CI 0.56–0.93). For SCC, weak evidence indicated that laser-assisted PDT results in a higher complete response rate than conventional PDT (RR 2.75, 95% CI 2.19–3.45). Analysis of data from mixed BCC and SCC populations provided weak evidence that complete response rate to MAL-PDT at three months was higher than that to placebo (RR 2.76, 95% CI 2.65–2.86) but lower than those to erbium:yttrium-aluminum-garnet ablative fractional laser (YAG-AFL)-PDT at 3 months and 1 year. Furthermore, laser-assisted PDT was associated with a higher complete response rate than conventional PDT (RR 0.38, 95% CI 0.17–0.82).

Thirteen associations with recurrence were identified (**Figure 3B**; **Supplementary Table S5**). In BCC, weak evidence suggested that PDT led to higher 1-year (RR 1.82, 95% CI 1.52–2.18) and 2-year (RR 1.95, 95% CI 1.38–2.75) recurrence rates than surgery, with no difference in 3 to 5-year recurrence rates. Additionally, the 1-year recurrence rate for MAL-PDT was higher than that for other therapies (RR 1.38, 95% CI 1.07–1.77). Further, weak evidence indicated that YAG-AFL-PDT leads to a lower 1-year recurrence rate in mixed BCC and SCC populations (RR 2.56, 95% CI 1.47–4.47) and a lower two-year recurrence rate in SCC populations (RR 3.42, 95% CI 1.52–7.69) than MAL-PDT.

Regarding cosmetic outcomes, five associations were identified (**Figure 3C**; **Supplementary Table S5**). For BCC, no difference in cosmetic outcomes between MAL-PDT and other therapies was detected, while for SCC, there was weak evidence that MAL-PDT results in better cosmetic outcomes than cryotherapy (RR 3.46, 95% CI 1.55-7.73), but no difference was detected compared to 5-fluorouracil.

Twelve associations related to AEs and pain were identified (**Figure 3D**; **Supplementary Table S5**). In BCC, weak evidence indicated that AEs were slightly more frequent in patients treated with MAL-PDT compared to those undergoing other therapies (RR 1.47, 95% CI 1.02–2.11); MAL-PDT was associated with a higher risk of pain than placebo, 5-fluorouracil, and imiquimod. In SCC, weak evidence suggested that 5-Aminolevulinic acid-based PDT (ALA-PDT) led to a lower risk of pain than cryotherapy (RR 0.41, 95% CI 0.24–0.68) but not 5-fluorouracil; no difference in risk of AEs for MAL-PDT was detected compared to those for cryotherapy and 5-fluorouracil. In mixed BCC and SCC populations, no difference in risk of AEs was detected between MAL-PDT compared to placebo and YAG-AFL-PDT.

For Bowen’s disease, weak evidence suggested that PDT leads to a higher lesion reduction rate than 5-fluorouracil and cryotherapy (RR 3.19, 95% CI 1.23–8.29), but no differences were detected in recurrence or complete response rates (**Figure 3E**; **Supplementary Table S5**).

### 3.5 Evidence related to PDT from single arm meta-analysis

**Figure 4:**
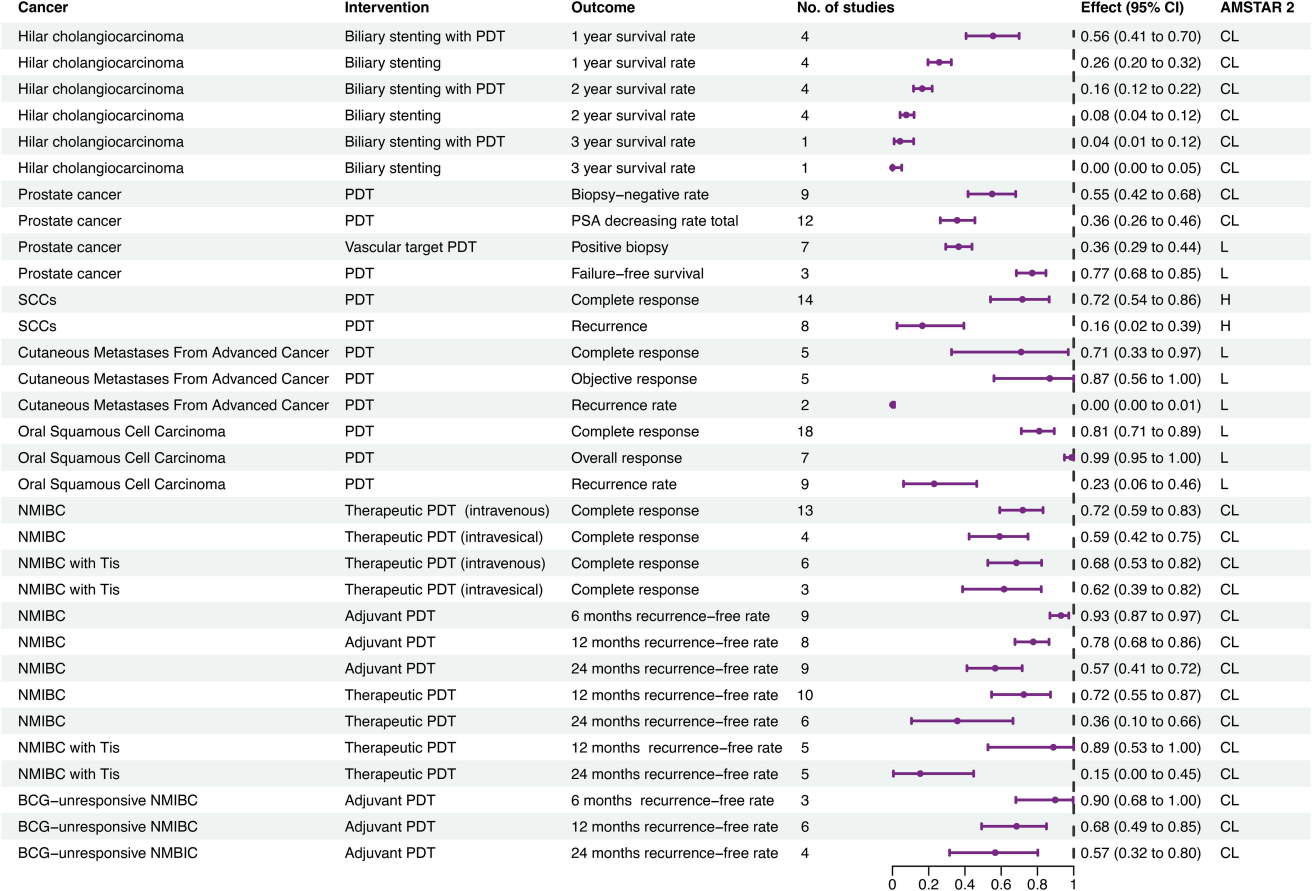
Forest plot of single arm meta-analyses. AMSTAR 2, assessment of multiple systematic reviews; BCG, Bacille Calmette-Guérin; CL, critical low; CI, confidence interval; ES, effect size; H, high; L, low; NMIBC, nonmuscle invasive bladder cancer; PDT, photodynamic therapy; SCC, squamous cell carcinoma.

Thirty-two associations with single-arm outcomes were detected (**Figure 4**; **Supplementary Table S6**). For hilar cholangiocarcinoma, 1-, 2-, and 3-year survival rates for patients undergoing biliary stenting with PDT were 56%, 16%, and 4%, respectively, compared with 26%, 8%, and 0% for biliary stenting alone.

For prostate cancer, patients receiving PDT had a biopsy-negative rate of 55%, a prostate specific antigen decrease rate of 36%, and a failure-free survival rate of 77%, and the positive biopsy rate for vascular-targeted PDT was 36%.

For non-metastatic SCC of the skin, the complete response rate and recurrence rate were 14% and 16%, respectively. In patients with cutaneous metastases from advanced cancer, PDT had complete response, objective response, and recurrence rates of 71%, 87%, 0%, respectively, while in patients with oral SCC complete response, overall response, and recurrence rates were 81%, 99%, and 23%, respectively.

For non-muscle invasive bladder cancer (NMIBC), therapeutic PDT was associated with 1- and 2-year recurrence-free rates of 72% and 36%, respectively. Further, the complete response rate for therapeutic PDT with intravenous photosensitizer was 72%, while that for intravesical photosensitizer was 59%. Adjuvant therapeutic PDT led to 6-month, 1-year, and 2-year recurrence-free rates of 93%, 78%, and 57%, respectively. For NMIBC with tumor *in situ*, therapeutic PDT had 1- and 2-year recurrence-free rates of 89% and 15%, respectively. The complete response rate for therapeutic PDT with intravenous photosensitizer was 68%, while that for intravesical photosensitizer was 62%. For Bacillus Calmette-Guérin-unresponsive NMIBC, adjuvant PDT had 6-month, 1-year, and 2-year recurrence-free rates of 90%, 68%, and 57%, respectively.

### 3.6 Subgroup analysis

We pooled 56 associations for subgroup analysis, based on cancer type, study design, and control treatment (**Supplementary Table S7**).

Subgroup analysis by study design revealed that non-randomized controlled trials (RCTs) showed weak evidence that PDT extended OS for patients with unresectable cholangiocarcinoma (HR 0.67, 95% CI 0.52–0.85); however, no significant results were detected in RCTs.

Compared to imiquimod, MAL-PDT for BCC resulted in higher 1-year (RR 1.31, 95% CI 1.07–1.61), 3-year (RR 1.91, 95% CI 1.52–2.39), and 5-year (RR 1.46, 95% CI 1.21–1.77) recurrence rates, and a lower 1-year complete response rate (RR 0.74, 95% CI 0.55–0.98).

MAL-PDT for BCC also had higher 1-year (RR 1.85, 95% CI 1.32–2.58), 2-year (RR 1.82, 95% CI 1.30–2.55), and 5-year (RR 1.70, 95% CI 1.13–2.58) recurrence rates, and a higher risk of AEs (RR 1.64, 95% CI 1.19–2.27), than surgery. Further, MAL-PDT led to a lower 3-month complete response rate (RR 0.56, 95% CI 0.42–0.74), but better cosmetic outcomes (RR 3.99, 95% CI 2.44–6.51), than surgery. Similarly, ALA-PDT led to higher 1-year (RR 2.00, 95% CI 1.30–3.07) and 3-year (RR 2.18, 95% CI 1.74–2.74) recurrence risks than surgery.

Relative to cryotherapy, MAL-PDT for BCC led to a lower complete clearance rate (RR 0.70, 95% CI 0.52–0.95) but better cosmetic outcomes (RR 2.90, 95% CI 1.80–4.66), while MAL-PDT had higher complete clearance (RR 2.750, 95% CI 1.85–4.10), 3-month complete response (RR 2.75, 95% CI 1.85–4.10), and AEs (RR 2.72, 95% CI 1.31–5.63) rates than placebo.

### 3.7 Overlapping associations

Calculation of the CCA led to exclusion of 10 overlapping associations (**Supplementary Table S8**). Among these, two excluded associations indicated no significant difference in cosmetic outcomes between patients undergoing PDT and those receiving surgery or cryotherapy, which was inconsistent with included associations.

### 3.8 Publication bias

Funnel plot asymmetry tests were conducted for meta-analyses including at least ten studies. Since all paired meta-analyses included fewer than ten studies, funnel plots were not created. For twelve associations with an Egger’s test *P*-value < 0.1, the “trim-and-fill” method was used to adjust the effect size and 95% CI values, resulting in three associations losing significance (**Supplementary Table S9**). In single-arm meta-analyses, six associations included at least ten studies, and the funnel plots generated appeared roughly symmetrical (**Supplementary Figure S2**).

## 4 Discussion

The aim of this review was to investigate the evidence for associations between PDT and its efficacy and AEs in cancer treatment. We integrated data from 18 publications covering 8 types of cancer, involving 124 associations from paired meta-analyses and 34 associations from single-arm meta-analyses.

Cholangiocarcinoma is a rare, aggressive cancer originating from the bile ducts, for which PDT has emerged as a promising palliative treatment option (40). Due to the anatomical location and frequent late diagnosis of cholangiocarcinoma, treatment is challenging (41). Biliary stenting is commonly used for conservative treatment of unresectable cholangiocarcinoma, and addition of PDT may improve survival rates by reducing stent occlusion. Relative to biliary stenting alone, combined PDT significantly improves OS and reduces mortality by approximately 37% (RR 0.63, 95% CI 0.48–0.83), without increasing the risk of AEs; however, when only RCT studies were considered, the improvement in OS with PDT was not significant, suggesting potential reporting or publication bias. For hilar cholangiocarcinoma, re-synthesized meta-analysis showed that PDT combined with biliary stenting does not improve OS, indicating that previous effects may have been overestimated. This finding suggests that cancer type may influence PDT efficacy; the differences could be due to the anatomical complexity and aggressiveness of hilar cholangiocarcinoma, which pose significant challenges for effective PDT delivery and tumor eradication. Overall, these results indicate that, while PDT holds great potential for use against certain cholangiocarcinoma subtypes, its efficacy may be limited for others.

Previous meta-analyses have provided extensive evidence of the efficacy of PDT for non-melanoma skin cancers, particularly BCC and SCC. While surgical excision remains the gold standard for many melanoma skin cancers, particularly high-risk or aggressive lesions, PDT offers a non-invasive alternative that preserves tissue integrity and function, which is especially important for lesions on the face, ears, and other visible areas (42). Multiple guidelines recommend considering PDT for superficial or nodular BCC with small diameter (< 2 cm) and thin lesions (< 2 mm) in patients unsuitable for surgery (43–45). PDT has excellent cosmetic outcomes in patients with BCC, but lower complete response rates and higher recurrence rates than surgery and imiquimod. PDT also carries a higher risk of pain (15%–70%) and AEs than placebo, fluorouracil, and imiquimod, and a 64% higher risk of AEs than surgery; however, after accounting for publication bias using the trim-and-fill method, the risk of AEs associated with PDT relative to various other therapies lost significance, suggesting the presence of publication bias and potential biases due to heterogeneous comparisons.

Our quantitative analysis substantiates the cosmetic advantages of PDT, particularly in head-to-head comparisons with conventional therapies. The pooled data demonstrated that MAL-PDT achieved 3.46-fold better cosmetic outcomes than cryotherapy in SCC (RR 3.46, 95% CI 1.55-7.73), and maintained 2.90- to 3.99-fold superiority over both cryotherapy (RR 2.90) and surgery (RR 3.99) in BCC management. This magnitude of effects likely stems from PDT’s tissue-sparing mechanism: selective photosensitizer activation minimizes collagen disruption and preserves dermal architecture (46), whereas surgical excision inherently causes structural defects and cryotherapy induces collagen hyalinization (47). Notably, the absence of cosmetic difference between MAL-PDT and 5-fluorouracil suggests that non-invasive pharmacological approaches may share similar aesthetic preservation profiles. However, long-term cosmetic outcomes beyond 5 years remain unquantified, particularly regarding pigmentary changes. The trade-off between recurrence risk (RR 1.82 vs surgery) and cosmetic superiority necessitates shared decision-making, especially for high-risk tumors where oncologic control takes precedence.

PDT is generally well-tolerated, with side effects typically less severe than those from traditional treatments, such as surgery or radiation therapy (48). Common side effects include local skin reactions, such as erythema, edema, and pain at the treatment site, are usually mild to moderate, and generally resolve within days to weeks after treatment. Photosensitivity is a significant concern, necessitating strict light protection measures, to avoid adverse reactions (49).

In SCC populations, current evidence indicates that PDT results in comparable recurrence and complete response rates to cryotherapy and fluorouracil, but offers superior cosmetic outcomes relative to cryotherapy. Additionally, there is evidence that laser-assisted PDT is superior to conventional PDT in achieving complete response and reducing recurrence, without increasing the risk of AEs; however, after correcting for publication bias, the complete response rate of laser-assisted PDT does not differ significantly from that of conventional PDT. Furthermore, our analysis systematically compares the efficacy and safety profiles of distinct photosensitizers. Current evidence demonstrates no significant differences among ALA, MAL, and hexaminolevulinic acid in clinical outcomes. Notably, laser-assisted PDT using YAG-AFL-PDT shows potential superiority over MAL-PDT in both therapeutic response and recurrence reduction, while maintaining equivalent cosmetic outcomes and adverse event risks. This suggests that technological refinements in PDT delivery systems – rather than photosensitizer selection alone – may enhance therapeutic performance. Emerging clinical trials (ClinicalTrials.gov identifiers: NCT05374915, NCT02840331, NCT02367547, NCT06262555) are actively evaluating combinatorial PDT approaches with novel light sources and photosensitizer formulations. Future validation through network meta-analyses will be essential to delineate hierarchical efficacy patterns across PDT modalities once more comprehensive datasets from these studies become available.

Single-arm meta-analyses suggest that the relative efficacy of PDT varies across different cancers. For example, PDT demonstrates high efficacy and low recurrence rates in patients with cutaneous metastatic SCC and oral SCC, whereas it is less effective in primary cutaneous SCC and hilar cholangiocarcinoma. This variation may be influenced by the depth, organ, and nature of lesions. In addition, PDT has shown considerable therapeutic promise in prostate and bladder cancer, but higher levels of clinical evidence are needed (50, 51).

The three key elements influencing PDT efficacy are the photosensitizer, exposure to specific wavelengths of light, and oxygen (52, 53). Photosensitizers can be administered via intravenous injection or topical application, after which they accumulate in tumor tissue, due to characteristics such as leaky vasculature and an acidic environment, resulting in higher concentrations in tumor than in normal tissue (54). This selective accumulation helps minimize toxicity to normal tissue and enhances cancer specificity (55).

**Figure 5:**
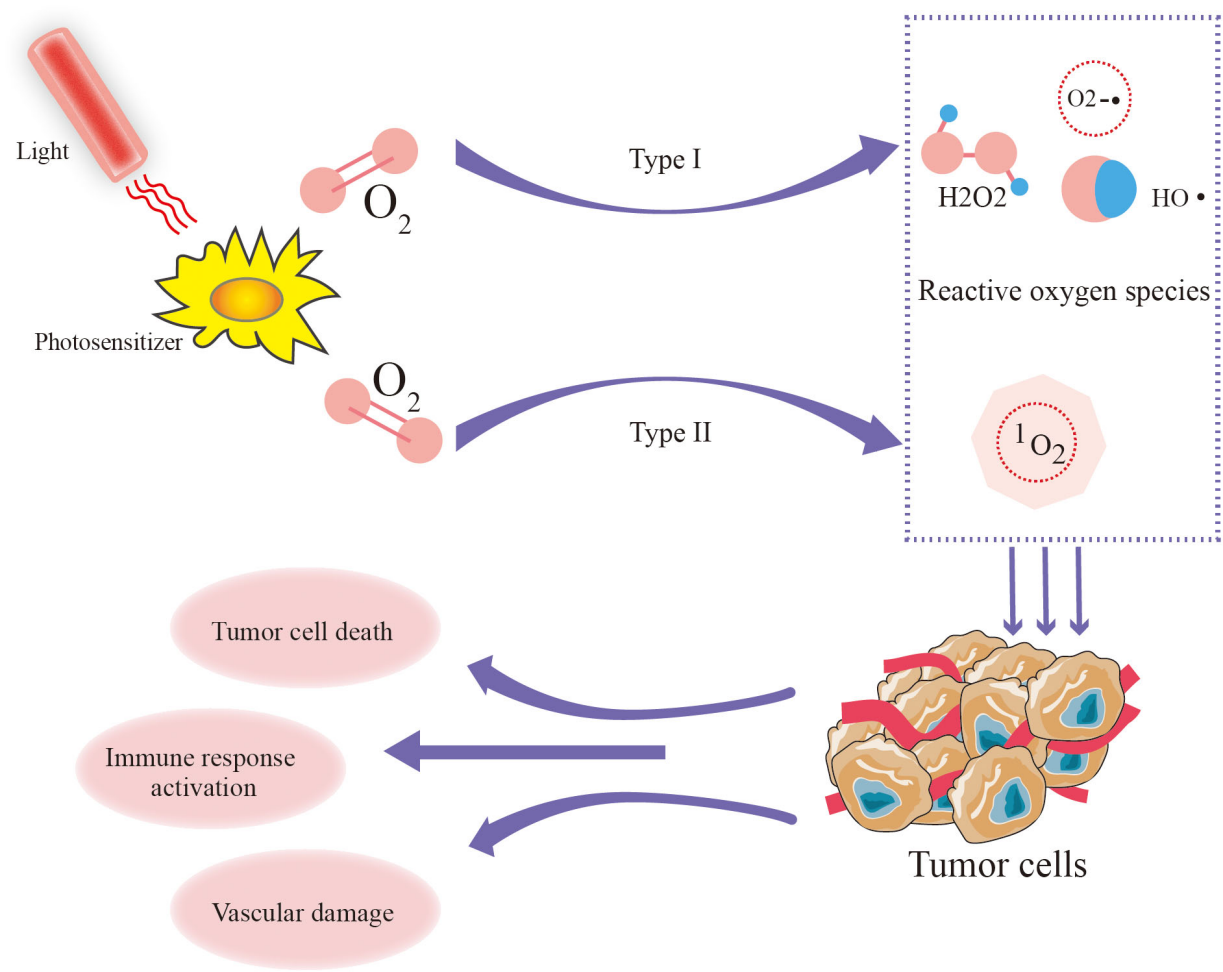
The role and mechanism of photodynamic therapy in tumors.

Under the influence of the photosensitizer, energy from light is transferred to molecular oxygen, producing reactive oxygen (ROS) including singlet oxygen (^1^O_2)_, superoxide radicals (O2−•), hydroxyl radicals (HO•), and hydrogen peroxide (H_2_O_2_) (56, 57). PDT efficacy primarily relies on the generation of ¹O_2_ (58), unlike molecular oxygen, which plays a passive role in cellular metabolism, has a short lifespan and exerts direct cytotoxic effects by damaging cellular components, including lipids, proteins, and DNA (59). The amount of ¹O_2_ produced is a crucial determinant of PDT efficacy and is influenced by the type of photosensitizer, its subcellular localization, oxygen availability, and light fluence (60) (**Figure 5**). Notably, recent research on porphyrin β - thiolation shows that progressive thiolation can switch ^1^O_2_ photosensitization, adding a new dimension to understanding the factors affecting ¹O_2_ production in PDT (61).

Several strategies have been developed to enhance PDT efficacy by boosting ¹O_2_ generation and improving light penetration. Metallic nanoparticles, such as gold and titanium dioxide, can amplify ROS production via plasmon resonance and electron transfer (62, 63). To address tumor hypoxia, oxygen-delivering nanocarriers like perfluorocarbons have been introduced to sustain ¹O_2_ output (64). Meanwhile, near-infrared light sources (700–800 nm) enable deeper tissue penetration and improved therapeutic outcomes (65, 66). In addition, rational metal selection within photosensitizer complexes has emerged as an effective approach to modulate excited-state energy dissipation, thereby enhancing both photodynamic and photothermal therapeutic performance (67).

The pharmacokinetics of photosensitizers significantly impact PDT efficacy and safety (68). Most photosensitizers undergo hepatic metabolism and are eliminated via biliary or renal excretion (69, 70). Prolonged retention in healthy tissues, particularly the skin, may lead to photosensitivity-related toxicities (71). The development of tumor-targeting photosensitizers, such as those conjugated with antibodies, peptides, or folic acid, aims to improve tumor selectivity and minimize off-target effects (72–74). Enhanced permeability and retention effects in tumors facilitate the accumulation of macromolecular photosensitizers, further improving PDT specificity (75). Recently, bioinspired β-pyrrolic ring-opening seco-chlorins such as ZnBPL have been developed, which not only exhibit strong ROS generation and therapeutic efficacy but also undergo rapid metabolism, thereby reducing phototoxic side effects, representing a novel approach to enhance both the efficacy and safety of PDT (76).

Despite its selective tumor targeting, PDT is associated with various toxicities primarily related to photosensitizers. ROS can damage normal tissues, stimulate local immune responses, release inflammatory factors, induce oxidative stress, and trigger the release of cytokines, such as tumor necrosis factor-alpha, nitric oxide, histamine, and prostaglandin E2 (71), resulting in local inflammation and nerve ending stimulation. these inflammatory reactions also function in tissue repair, debris clearance, and internal balance restoration (77). Common adverse effects include local erythema, edema, and pain at the treatment site, which usually resolve within days to weeks (48). Strategies to mitigate these toxicities include dose optimization, light dose fractionation, and the development of rapidly clearing photosensitizers with improved photophysical properties (78, 79).

Factors affecting PDT efficacy include photosensitizer type, its specific localization in tumor tissue, light penetration depth, and local oxygen content (80). Porphyrin-based photosensitizers, which have a tetrapyrrole structure, are commonly used in cancer treatment, and should ideally have absorption peaks in the range 600–800 nm; light absorption and penetration are poor below 600 nm, while above 800 nm, light cannot excite oxygen molecules to singlet oxygen (81). Increasing wavelength enhances light penetration into tissues. Light source parameters and characteristics, including coherence, wavelength, and beam size, also influence PDT efficacy (82). The findings of our research indicate that laser light (a coherent light source) was slightly more effective in treating skin cancer than non-coherent light sources, and did not increase AEs. This may be because coherent light is monochromatic and can match the absorption peak of the photosensitizer, providing stronger penetration ability (83).

Combined PDT with chemotherapy, radiotherapy, or surgery demonstrates enhanced antitumor efficacy through synergistic mechanisms without increasing adverse events. For instance, doxorubicin-PDT combinations halve chemotherapy doses yet enhance cytotoxicity in breast cancer cells by overcoming multidrug resistance (84). This principle of localized oxidative damage amplification parallels recent advances in antibacterial chemodynamic therapy, where Fenton reaction-generated hydroxyl radicals synergize with photodynamic approaches to overcome biofilm resistance through microenvironment-specific ROS generation (85). Recent advances in photoimmunotherapy (PIT) further enhance therapeutic precision by leveraging antibody-targeted phototoxicity (Cetuximab-IR700 conjugates) and systemic immune activation, as evidenced by NK cell-mediated indocyanine green (ICG) delivery systems that synergize photothermal ablation with perforin/granzyme-dependent cytotoxicity (86, 87). Nanoparticle-mediated co-delivery of photosensitizers and chemotherapeutics (e.g., chlorin e6 with artesunate) leverages tumor-selective accumulation and controlled ROS-triggered drug release, improving therapeutic indices (88). This parallels PIT strategies utilizing nanocarriers (ICG-liposomes) to stabilize photosensitizers while exploiting immune cells (NK-92MI) as tumor-targeting vehicles, as demonstrated in dual-mechanism platforms (86, 87). Preclinical studies highlight pH- or enzyme-sensitive linkers in prodrugs, enabling localized activation and reduced systemic toxicity (89). Clinically, endoscopic PDT combined with gemcitabine/oxaliplatin for unresectable cholangiocarcinoma extends median survival by 1.5-fold versus monotherapies, with no added toxicity (40). PDT also radiosensitizes tumors: sequential PDT and radiotherapy for esophageal or lung cancer yield higher complete response rates via non-overlapping mechanisms (e.g., ROS-induced hypoxia enhancement) (90). Emerging strategies include PARP inhibitors (olaparib) with PDT, which lower light doses (25 vs. 100 J/cm²) while maintaining efficacy in gastric cancer models (91). These combinations exploit PDT’s spatial precision and immune modulation to amplify conventional therapies, underscoring their translational potential. Thus, rational pairing of PDT with chemotherapy, nanotechnology, or targeted agents offers a paradigm for improving survival without compromising safety. Notably, copper sulfide-based nanoplatforms exemplify this synergy by integrating photothermal ablation with chemotherapy and dynamic therapies, while enabling multimodal imaging-guided treatment to optimize tumor microenvironment modulation and minimize systemic toxicity (92).

To our knowledge, this is the first umbrella review to assess associations of PDT with cancer treatment, with a focus on evaluating methodological quality and evidence grades of relevant publications. We employed the CCA method to prevent duplication across studies and conducted a comprehensive meta-analysis to assess the current landscape. Additionally, we conducted a reanalysis using a random-effects model to identify and address publication bias, thereby strengthening the reliability of our results.

Nevertheless, our umbrella review has several important limitations that warrant consideration. First, most of the included studies were rated as having low or very low methodological quality. This was primarily due to inadequate reporting practices—including the lack of comprehensive literature lists, absence of pre-registration for study protocols, and insufficient assessment of bias risks—which collectively diminish the confidence in the reported findings. To address potential publication bias arising from these shortcomings, we conducted Egger’s test and generated funnel plot visualizations. Second, although subgroup analyses can provide insights into heterogeneity, our ability to perform such analyses based on key variables such as PDT dose, treatment duration, and ethnicity was constrained by the limitations inherent in the source meta-analyses. Where possible, we focused our subgroup analyses on the specific photosensitizers used and the treatment modalities applied in the control groups. However, this approach may not fully capture the influence of other clinically relevant factors. Third, the overall modest sample size across the studies contributes to the relatively low evidence grade (Class IV, weak evidence) observed in our review. This suggests that the results need to be interpreted with caution, in conjunction with other high-quality evidence or expert consensus. This limitation highlights the urgent need for larger, rigorously designed clinical trials—particularly randomized controlled trials—to further validate and expand upon our findings. Future studies should focus on optimizing PDT protocols, exploring new photosensitizers, and improving light delivery technologies, to enhance efficacy. Combination regimens require systematic investigation, including synergies with immune checkpoint inhibitors, hypoxia-activated prodrugs, and nanoparticle-mediated co-delivery systems to enhance therapeutic specificity. Multicenter consortia should establish standardized endpoints encompassing complete response rates, immune microenvironment modulation, and long-term recurrence metrics, while parallel cost-effectiveness analyses will be crucial for clinical translation. Personalized treatment approaches, based on genetic and molecular profiles, may also facilitate identification of patients most likely to benefit from PDT (68, 93) and prioritize robust methodological standards and comprehensive reporting to enhance the reliability of the evidence base for PDT in cancer treatment.

## 5 Conclusion

Current evidence indicates that PDT combined with stenting and chemotherapy in the treatment of cholangiocarcinoma decreases overall mortality and enhances OS. For patients with BCC and SCC, PDT results in higher recurrence rates than surgery, cryotherapy, and imiquimod, yet it yields superior cosmetic outcomes. Moreover, laser-based PDT demonstrates superior efficacy compared with conventional PDT. Overall, PDT shows promise for the treatment of prostate, oral, and bladder cancers.

PDT represents a versatile and evolving modality in cancer therapy, offering selective tumor targeting, cosmetic advantages, and potential synergy with novel technologies. However, efficacy varies across cancer types and is influenced by complex interactions among photosensitizers, light parameters, and tumor biology. Standardization of treatment protocols, development of next-generation photosensitizers, and high-quality RCTs across diverse indications are essential to establish PDT as a mainstream oncologic intervention.

## Data Availability

The original contributions presented in the study are included in the article/**Supplementary Material**. Further inquiries can be directed to the corresponding authors.

## Supplementary material

Supplementary Figure 1 AMSTAR 2 quality appraisal scores. AMSTAR, assessment of multiple systematic reviews. CDW, critical domains weaknesses (negatively answered); CL, critically low; L, low.

Supplementary Figure 2 Funnel plots for publication bias of single-arm meta-analyses. **(A)** complete response rate of SCC treated with PDT; **(B)** Complete response rate for PDT (intravenous) treatment of NMIBC; **(C)** 1-year recurrence rate of NMIBC treated with therapeutic PDT; **(D)** Complete response rate of oral SCC treated with PDT; **(E)** PSA reduction rate in PDT treatment of prostate cancer. Abbreviation: SCC, squamous cell carcinoma; NMIBC, nonmuscle invasive bladder cancer; PDT, photodynamic therapy; PSA, prostate specific antigen.

Supplementary Table 1 Nearly 10 years of clinical trials involving PDT treatment of cancer.

Supplementary Table 2 Detailed search strategies and results for PubMed.

Supplementary Table 3 List of excluded studies and reasons for their exclusion.

Supplementary Table 4 The photosensitizers and parameters used for different cancers and representative studies.

Supplementary Table 5 Detailed results of included and excluded associations.

Supplementary Table 6 Detailed results of the association in single arm meta-analyses.

Supplementary Table 7 Detailed results of subgroup meta-analyses association.

Supplementary Table 8 Citation matrices for meta-analyses with overlapping associations.

Supplementary Table 9 Recalculated detailed results of meta-analyses using the by trim-and-fill method.
