# Supplementary figures and images for: Umbrella review of photodynamic therapy for cancer: efficacy, safety, and clinical applications
Source: Front Oncol. 2025 Aug 4;15:1528314. doi: 10.3389/fonc.2025.1528314 (PMC12358287; doi:10.3389/fonc.2025.1528314)

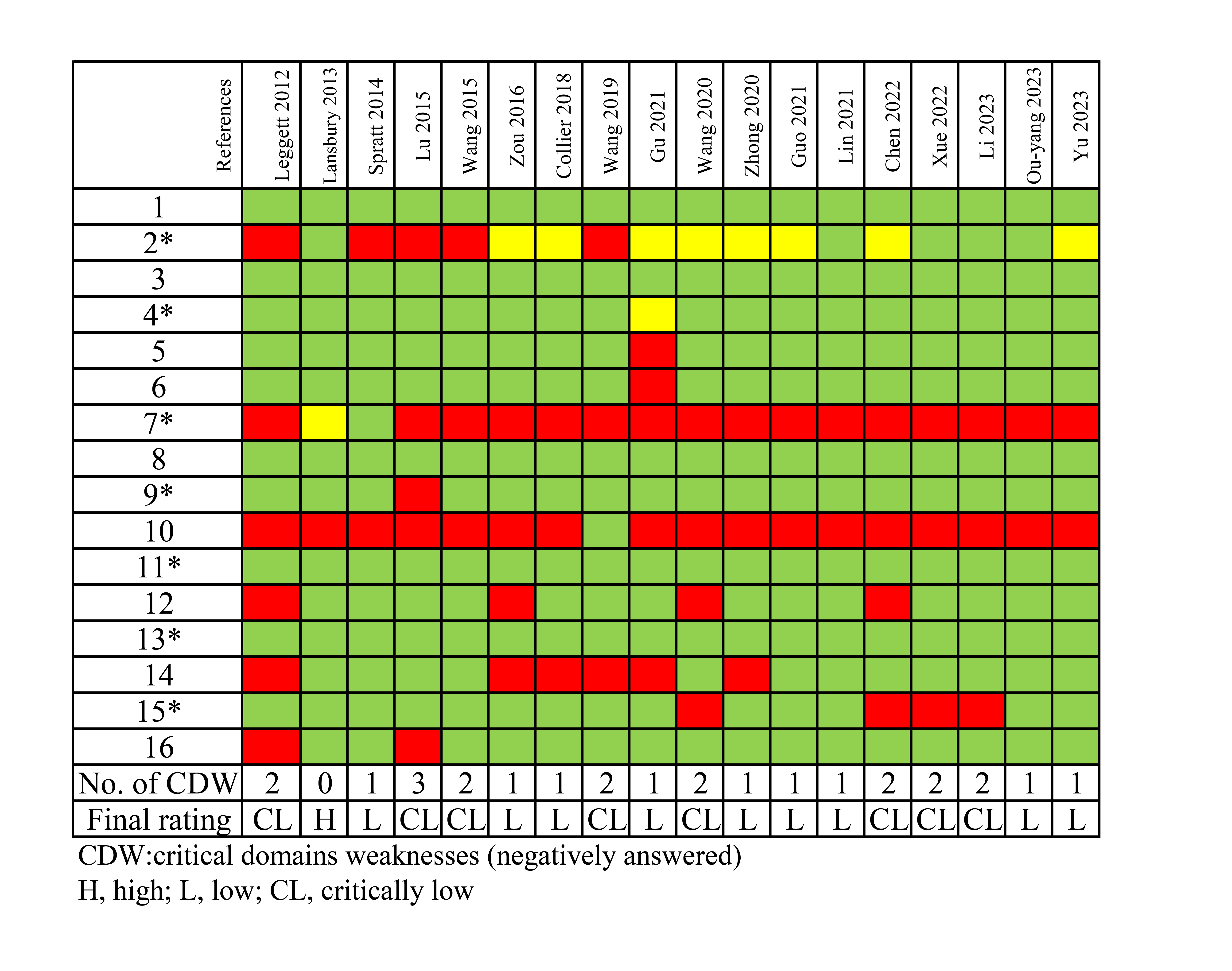

Supplement: Supplementary Figure 1 — AMSTAR 2 quality appraisal scores. AMSTAR, assessment of multiple systematic reviews. CDW, critical domains weaknesses (negatively answered); CL, critically low; L, low. [file Image1.tif]

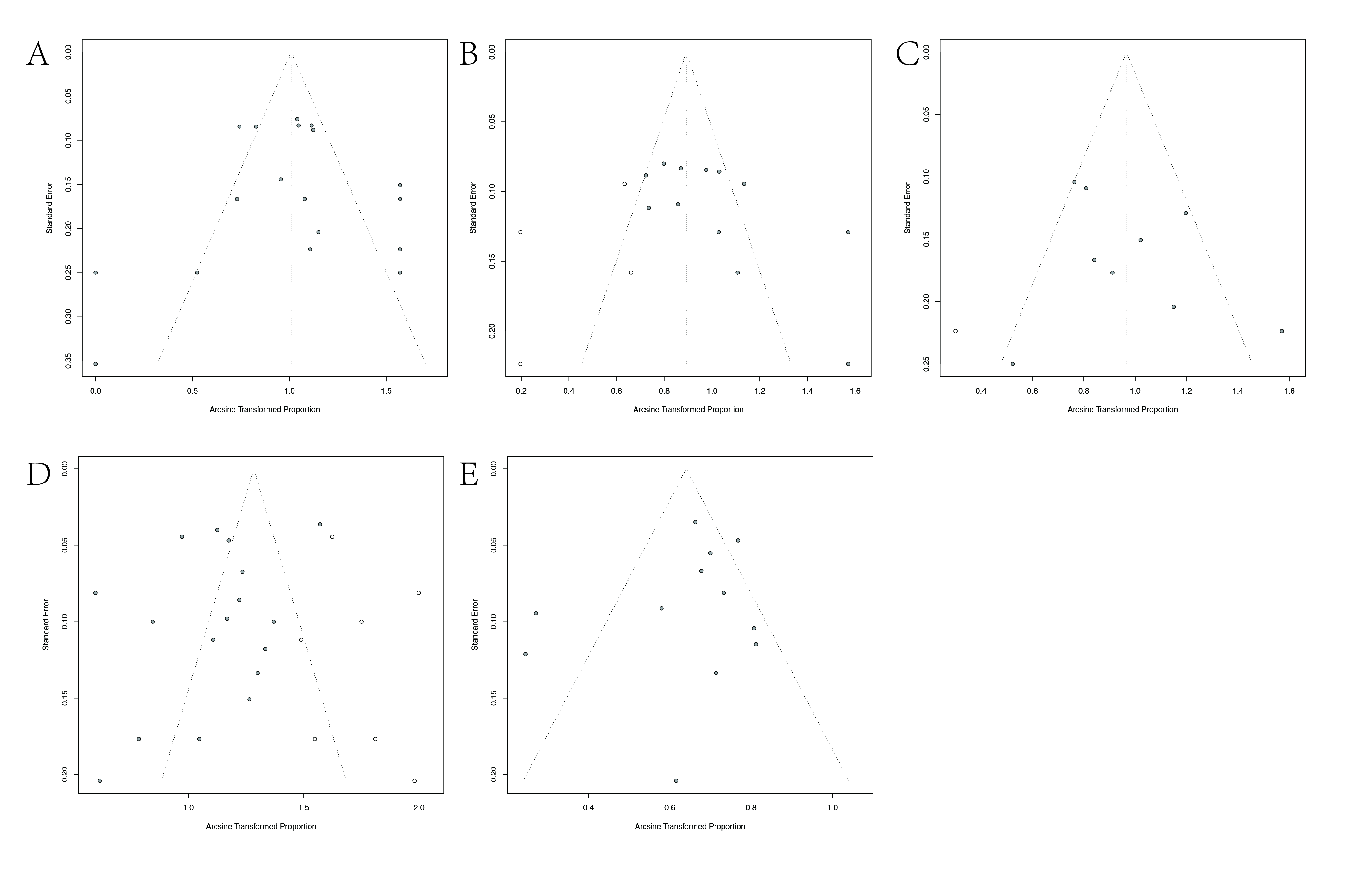

Supplement: Supplementary Figure 2 — Funnel plots for publication bias of single-arm meta-analyses. (A) complete response rate of SCC treated with PDT; (B) Complete response rate for PDT (intravenous) treatment of NMIBC; (C) 1-year recurrence rate of NMIBC treated with therapeutic PDT; (D) Complete response rate of oral SCC treated with PDT; (E) PSA reduction rate in PDT treatment of prostate cancer. Abbreviation: SCC, squamous cell carcinoma; NMIBC, nonmuscle invasive bladder cancer; PDT, photodynamic therapy; PSA, prostate specific antigen. [file Image2.tif]
