# Supplementary material for: Umbrella review of photodynamic therapy for cancer: efficacy, safety, and clinical applications
Source: Front Oncol. 2025 Aug 4;15:1528314. doi: 10.3389/fonc.2025.1528314 (PMC12358287; doi:10.3389/fonc.2025.1528314)
Supplement: Supplementary Table 1 — Nearly 10 years of clinical trials involving PDT treatment of cancer. [file Table1.docx]

Table S1. Nearly 10 years of clinical trials involving PDT treatment of cancer.

| **Photosensitizer** | **Cancer** | **Phase** | **Status** | **Country** | **Reference** | **Study start** |
| --- | --- | --- | --- | --- | --- | --- |
| Porfimer sodium | Malignant mesothelioma;Non-small cell lung carcinoma withpleural disorder | I | Recruiting | USA | NCT03678350 | Sep-21 |
|  | Non-small cell lung cancer;Lung metastasis | Early I | Unknown | Taiwan | NCT04753918 | Mar-21 |
|  | Locally advanced lung carcinoma; Non-small cell lung carcinoma; Small cell lung carcinoma; Lung Cancer AJCC v8 (Stage III/IIIA/IIIB/IIIC) | I/II | Recruiting | USA | NCT03735095 | Feb-20 |
|  | Non-small cell lung cancer | N/A | Terminated | USA | NCT03564054 | Oct-18 |
|  | Lung cancer and metastasis | I | Completed | USA | NCT03344861 | Aug-17 |
|  | Non-small cell lung cancer;Lung Metastasis | I | Completed | USA, Canada | NCT02916745 | Jan-17 |
|  | Malignant pleural mesothelioma | II | Completed | France | NCT02662504 | Jan-16 |
|  | Transbronchial photodynamic therapy for ablation of peripheral lung cancer | N/A | Recruiting | Taiwan | NCT06262555 | Mar-24 |
|  | Esophageal adenocarcinoma (Stage I, II, III); Esophageal cell carcinoma (Stage I, II, III) | III | Unknown | China | NCT02628665 | Oct-15 |
|  | Recurrent high-grade gliomas | II | Terminated | USA | NCT01966809 | Jun-15 |
|  | Hilar cholangiocarcinoma | III | Terminated | USA | NCT02082522 | Nov-14 |
|  | Epithelioid malignant pleural mesothelioma | II | Recruiting | USA | NCT02153229 | May-14 |
|  | Head and neck | II | Terminated | USA | NCT02068157 | Apr-14 |
|  | Advanced rectal cancer | II/III | Suspended | China | NCT01872104 | Aug-13 |
|  | Cholangiocarcinoma | N/A | Suspended | China | NCT01859169 | Jun-13 |
|  | Acinar cell adenocarcinoma of the pancreas; Duct cell adenocarcinoma of the pancreas; Pancreatic Cancer (Stage III) | I | Completed | USA | NCT01770132 | Apr-13 |
|  | Recurrent pediatric brain tumor | I | Completed | USA | NCT01682746 | Mar-13 |
| Hematoporphyrin | Cholangiocarcinoma | N/A | Not yet recruiting | China | NCT05580328 | Dec-22 |
|  | Esophageal carcinoma in Situ AJCC V7 | N/A | Not yet recruiting | China | NCT05208775 | Mar-22 |
|  | Cholangiocarcinoma non-resectable | N/A | Recruiting | China | NCT04860154 | Apr-21 |
|  | Recurrent or residual superficial esophageal carcinoma | N/A | Not yet recruiting | China | NCT06437288 | May-24 |
| Polyhematoporphyrin | Hilar cholangiocarcinoma | N/A | Completed | Austria | NCT02504957 | Jul-15 |
| Verteporfin | Recurrent prostate cancer | I/II | Recruiting | USA, Canada, UK | NCT03067051 | Mar-17 |
|  | Advanced pancreatic carcinoma; Locally advanced pancreatic carcinoma; Metastatic pancreatic carcinoma;Pancreatic neoplasm;Pancreatic carcinoma;Pancreatic cancer AJCC v8 unresectable (Stage II, IIA, IIB, III, IV) | II | Recruiting | USA | NCT03033225 | Dec-16 |
|  | Metastatic breast cancer | II | Unknown | USA | NCT02939274 | Oct-16 |
| Deuteporfin | Cholangiocarcinoma | II | Terminated | China | NCT02955771 | May-17 |
| Temoporfin | Cholangiocarcinoma | II | Recruiting | China | NCT03003065 | Mar-14 |
|  | Recurrent non-small cell lung carcinoma(Stage IIA, IIB, IIIA, IIIB) | I | Completed | USA | NCT01854684 | Feb-14 |
| Chlorin e6 | Advanced hilar cholangiocarcinoma | II | Unknown | South Korea | NCT02725073 | Jan-16 |
| HPPH | Head and neck | II | Terminated | USA | NCT03090412 | May-18 |
|  | Esophageal cancer | I | Unknown | China | NCT03757754 | Jun-15 |
| LUZ11 | Head and neck cancer | I/II | Recruiting | Portugal | NCT02070432 | Feb-14 |
| Padeliporfin | Transitional cellcancer of renal pelvis and ureter | III | Recruiting | USA, Austria, France, Israel | NCT04620239 | Mar-21 |
|  | Low risk prostate cancer | IV | Terminated | France | NCT03849365 | Jan-19 |
|  | Upper tract urothelial carcinoma | I | Active, not recruiting | USA | NCT03617003 | Aug-18 |
|  | Intermediate risk prostate cancer | II | Active, not recruiting | USA | NCT03315754 | Oct-17 |
|  | Esophagogastric cancer with moderate to severe dysphagia | I | Completed | USA, Israel | NCT03133650 | Apr-17 |
|  | Renal cancer | I/II | Terminated | UK | NCT01573156 | May-13 |
|  | Localized prostate cancer | III | Completed | Mexico, Panama, Peru | NCT01875393 | Mar-13 |
|  | Unresectable pancreatic ductal adenocarcinoma | I | Recruiting | USA | NCT05919238 | May-24 |
|  | High risk of peripheral primary lung cancer, stage 1A1/1A2 | I | Recruiting | USA | NCT05918783 | May-25 |
| Photobac | Glioblastoma multiforme of brainglioma, sarcomatous | I | Not yet recruiting | USA | NCT05363826 | Nov-22 |
| Silicon phthalocyanine 4 | Recurrent cutaneous T-cell non-Hodgkin lymphoma; Recurrent mycosis fungoides/sezary syndrome; Cutaneous T-cell non-Hodgkin lymphoma (Stage I/IIA); Mycosis fungoides/sezary syndrome (Stage IA/IIA/IB) | I | Completed | USA | NCT01800838 | Apr-13 |
| TLD-1433 | Non-muscle invasivebladder cancerrefractory to BCG | II | Recruiting | USA, Canada | NCT03945162 | Aug-19 |
|  | Non-muscle invasivebladder cancer refractory to BCG | I | Completed | Canada | NCT03053635 | Dec-16 |
| Hypericin | Cutaneous T-cell lymphoma; Mycosis fungoides | II | Completed | USA | NCT05380635 | May-22 |
|  | Peritoneal carcinomatosis | III | Unknown | Germany | NCT02840331 | Jul-17 |
| REM-001 | Cutaneous metastatic breast cancer | II | Recruiting | USA | NCT05374915 | Feb-24 |
| 5-ALA | Non-muscle invasive bladder cancer | N/A | Not yet recruiting | China | NCT05547516 | Sep-22 |
|  | Skin tumors and non-cancer skin disorders | N/A | Recruiting | China | NCT05488860 | Jul-22 |
|  | Glioblastoma | II | Recruiting | Belgium, France | NCT04391062 | Sep-21 |
|  | Glioblastoma | II | Recruiting | Germany | NCT04469699 | Apr-21 |
|  | Superficial and nodular basal cell carcinoma | II | Recruiting | USA | NCT04552990 | Sep-20 |
|  | Basal cell carcinoma; Basal cell nevus syndrome | I | Active, not recruiting | USA | NCT03467789 | Oct-18 |
|  | Superficial basal cell carcinoma | III | Recruiting | USA | NCT03573401 | Sep-18 |
|  | Skin cancernon-melanoma; skin cancer sun damaged skin; Actinic keratoses | I/II | Recruiting | USA | NCT03110159 | Aug-17 |
|  | Glioblastoma | N/A | Completed | France | NCT03048240 | May-17 |
|  | Head and neck | I/II | Completed | India | NCT03638622 | Mar-17 |
|  | Squamous cell carcinoma | N/A | Unknown | USA | NCT03025724 | Jan-17 |
|  | Nonmelanoma skin cancers in organ transplant recipients | N/A | Completed | USA | NCT02751151 | Feb-16 |
|  | Multiple basal cell carcinoma | I | Not yet recruiting | USA | NCT06623201 | Dec-24 |
| 5-ALA and MAL | Basal cell carcinoma | I | Completed | USA | NCT02639117 | Nov-15 |
|  | Basal cell carcinoma | III | Completed | Germany | NCT02144077 | Jan-14 |
|  | Superficial basal cell carcinoma | IV | Unknown | Netherlands | NCT01491711 | Aug-13 |
| 5-ALA, MAL and HAL | Neoplasms; Basal cell carcinoma | I/II | Active, not recruiting | Finland | NCT02367547 | Mar-15 |
| MAL | Superficial basal cell carcinoma; Bowen’s Disease | N/A | Completed | Belgium | NCT03012009 | Sep-14 |
