# Supplementary material for: Umbrella review of photodynamic therapy for cancer: efficacy, safety, and clinical applications
Source: Front Oncol. 2025 Aug 4;15:1528314. doi: 10.3389/fonc.2025.1528314 (PMC12358287; doi:10.3389/fonc.2025.1528314)
Supplement: Supplementary Table 2 — Detailed search strategies and results for PubMed. [file Table2.docx]

Table S2. Detailed search strategies and results for PubMed.

| Search number | Query | Results |
| --- | --- | --- |
| #1 | Photodynamic Therapy OR photochemotherapy | 31,298 |
| #2 | Neoplasms [MeSH Terms] | 3,953,084 |
| #3 | Tumor OR Neoplasm OR Tumors OR Neoplasia OR Neoplasias OR Cancer OR Cancers OR Malignant Neoplasm OR Malignancy OR Malignancies OR Malignant Neoplasms OR Neoplasm, Malignant OR Neoplasms, Malignant OR Benign Neoplasms OR Benign Neoplasm OR Neoplasms, Benign OR Neoplasm, Benign | 3,550,530 |
| #4 | #2 OR #3 | 5,053,869 |
| #5 | "meta analysis" OR "meta analysis" OR "meta analys*" OR "meta analys*" OR "metaanalys*" OR "systematic review" OR "systematic literature review" OR "systematic overview" OR "quantitative review" OR "quantitative overview" OR "quantitative synthesis" OR "methodologic review" OR "methodologic overview" | 458,389 |
| 6 | #1 AND #4 AND #5 | 215 |
