# Supplementary material for: Umbrella review of photodynamic therapy for cancer: efficacy, safety, and clinical applications
Source: Front Oncol. 2025 Aug 4;15:1528314. doi: 10.3389/fonc.2025.1528314 (PMC12358287; doi:10.3389/fonc.2025.1528314)
Supplement: Supplementary Table 3 — List of excluded studies and reasons for their exclusion. [file Table3.docx]

Table S3. List of excluded studies and reasons for their exclusion.

| No. | First author | Year | Title | Reason for Exclusion |
| --- | --- | --- | --- | --- |
| 1 | D. E. Spratt | 2013 | Palliative local therapy for skin metastases: A meta-analysis [1] | Abstract |
| 2 | X. H. Tao | 2014 | Efficacy and safety of photodynamic therapy for cervical intraepithelial neoplasia: a systemic review [2] | No meta-analysis |
| 3 | E. W. Cerrati | 2015 | The efficacy of photodynamic therapy in the treatment of oral squamous cell carcinoma: a meta-analysis [3] | Incomplete data |
| 4 | F. Vohra | 2015 | Efficacy of photodynamic therapy in the management of oral premalignant lesions. A systematic review [4] | No meta-analysis |
| 5 | K. Kumagai | 2016 | Systematic review and meta-analysis on the significance of salvage esophagectomy for persistent or recurrent esophageal squamous cell carcinoma after definitive chemoradiotherapy [5] | No photodynamic therapy |
| 6 | R. R. Lv | 2017 | A Network Meta-Analysis of Non-Melanoma Skin Cancer (NMSC) Treatments: Efficacy and Safety Assessment [6] | Network meta-analysis |
| 7 | H. Moole | 2017 | Success of photodynamic therapy in palliating patients with nonresectable cholangiocarcinoma: A systematic review and meta-analysis [7] | Incomplete data |
| 8 | L. J. James | 2018 | Interventions for the prevention of skin cancers in solid organ transplant recipients: A systematic review of randomized controlled trials [8] | Abstract |
| 9 | W. Zhang | 2018 | Efficacy and safety of photodynamic therapy for cervical intraepithelial neoplasia and human papilloma virus infection: A systematic review and meta-analysis of randomized clinical trials [9] | Precancerous lesions |
| 10 | E. Y. M. Chung | 2019 | Interventions to Prevent Nonmelanoma Skin Cancers in Recipients of a Solid Organ Transplant: Systematic Review of Randomized Controlled Trials [10] | Aim at cancer prevention |
| 11 | C. Fu | 2019 | Comment on "Photodynamic therapy in the treatment of basal cell carcinoma: A systematic review and meta-analysis" [11] | Comment |
| 12 | T. Fukumoto | 2019 | Comparing treatments for basal cell carcinoma in terms of long-term treatment-failure: a network meta-analysis [12] | Network meta-analysis |
| 13 | L. J. James | 2020 | Behavioural and pharmaceutical interventions for the prevention of skin cancers in solid organ transplant recipients: A systematic review of randomised controlled trials [13] | Aim at cancer prevention |
| 14 | L. Li | 2020 | Comment on "Efficacy and safety of photodynamic therapy with amino-5-laevulinate nanoemulsion versus methyl-5-aminolaevulinate for actinic keratosis: A meta-analysis" [14] | Comment |
| 15 | Y. C. C. Liew | 2020 | Photodynamic therapy for the prevention and treatment of actinic keratosis/squamous cell carcinoma in solid organ transplant recipients: a systematic review and meta-analysis [15] | Aim at cancer prevention and precancerous lesions |
| 16 | G. Mpourazanis | 2020 | The effectiveness of photodynamic therapy and cryotherapy on patients with basal cell carcinoma: A systematic review and meta-analysis [16] | Incomplete data |
| 17 | I. Snast | 2020 | Nonsurgical Treatments for Extramammary Paget Disease: A Systematic Review and Meta-Analysis [17] | Incomplete data |
| 18 | A. Unanyan | 2021 | Efficacy of photodynamic therapy in women with HSIL, LSIL and early stage squamous cervical cancer: a systematic review and meta-analysis [18] | Precancerous lesions |
| 19 | A. Binnal | 2022 | Photodynamic therapy for oral potentially malignant disorders: A systematic review and meta-analysis [19] | Precancerous lesions |
| 20 | R. Choudhary | 2022 | The Effect of Photodynamic Therapy on Oral-Premalignant Lesions: A Systematic Review [20] | Precancerous lesions |
| 21 | A. M. C. Ibarra | 2022 | Photodynamic therapy for squamous cell carcinoma of the head and neck: narrative review focusing on photosensitizers [21] | No meta-analysis |
| 22 | S. Song | 2022 | Comparative efficacy and safety of local palliative therapeutics for unresectable malignant biliary obstruction: a Bayesian network meta-analysis [22] | Network meta-analysis |
| 23 | J. R. Stewart | 2022 | Efficacy of nonexcisional treatment modalities for superficially invasive and in situ squamous cell carcinoma: A systematic review and meta-analysis [23] | Incomplete data |
| 24 | T. Yongpisarn | 2022 | Durable Clearance Rate of Photodynamic Therapy for Bowen Disease and Cutaneous Squamous Cell Carcinoma: A Systematic Review and Meta-Analysis [24] | Incomplete data |
| 25 | D. G. Fornel | 2023 | Photodynamic Therapy Can Modulate the Nasopharyngeal Carcinoma Microenvironment Infected with the Epstein-Barr Virus: A Systematic Review and Meta-Analysis [25] | In vitro study |
| 26 | X. Yuan | 2023 | Network meta-analysis of treatments for perineal extramammary paget's disease: Focusing on performance of recurrence prevention [26] | Network meta-analysis |
| 27 | N. Shanazarov | 2024 | Evaluation of the effectiveness and safety of photodynamic therapy in the treatment of precancerous diseases of the cervix (neoplasia) associated with the human papillomavirus: A systematic review [27] | Precancerous lesions |

**Reference**

1. Spratt, D.E., et al., *Palliative local therapy for skin metastases: A meta-analysis.* International Journal of Radiation Oncology Biology Physics, 2013. 87(2): p. S91.

2. Tao, X.H., et al., *Efficacy and safety of photodynamic therapy for cervical intraepithelial neoplasia: a systemic review.* Photodiagnosis Photodyn Ther, 2014. 11(2): p. 104-12.

3. Cerrati, E.W., et al., *The efficacy of photodynamic therapy in the treatment of oral squamous cell carcinoma: a meta-analysis.* Ear Nose Throat J, 2015. 94(2): p. 72-9.

4. Vohra, F., et al., *Efficacy of photodynamic therapy in the management of oral premalignant lesions. A systematic review.* Photodiagnosis Photodyn Ther, 2015. 12(1): p. 150-9.

5. Kumagai, K., et al., *Systematic review and meta-analysis on the significance of salvage esophagectomy for persistent or recurrent esophageal squamous cell carcinoma after definitive chemoradiotherapy.* Diseases of the Esophagus, 2016. 29(7): p. 734-739.

6. Lv, R.R. and Q. Sun, *A Network Meta-Analysis of Non-Melanoma Skin Cancer (NMSC) Treatments: Efficacy and Safety Assessment.* Journal of Cellular Biochemistry, 2017. 118(11): p. 3686-3695.

7. Moole, H., et al., *Success of photodynamic therapy in palliating patients with nonresectable cholangiocarcinoma: A systematic review and meta-analysis.* World J Gastroenterol, 2017. 23(7): p. 1278-1288.

8. James, L.J., et al., *Interventions for the prevention of skin cancers in solid organ transplant recipients: A systematic review of randomized controlled trials.* Nephrology, 2018. 23: p. 20.

9. Zhang, W., et al., *Efficacy and safety of photodynamic therapy for cervical intraepithelial neoplasia and human papilloma virus infection: A systematic review and meta-analysis of randomized clinical trials.* Medicine (Baltimore), 2018. 97(21): p. e10864.

10. Chung, E.Y.M., S.C. Palmer, and G.F.M. Strippoli, *Interventions to Prevent Nonmelanoma Skin Cancers in Recipients of a Solid Organ Transplant: Systematic Review of Randomized Controlled Trials.* Transplantation, 2019. 103(6): p. 1206-1215.

11. Fu, C., et al., *Comment on "Photodynamic therapy in the treatment of basal cell carcinoma: A systematic review and meta-analysis".* Photodiagnosis Photodyn Ther, 2019. 28: p. 132.

12. Fukumoto, T., et al., *Comparing treatments for basal cell carcinoma in terms of long-term treatment-failure: a network meta-analysis.* J Eur Acad Dermatol Venereol, 2019. 33(11): p. 2050-2057.

13. James, L.J., et al., *Behavioural and pharmaceutical interventions for the prevention of skin cancers in solid organ transplant recipients: A systematic review of randomised controlled trials.* BMJ Open, 2020. 10(5).

14. Li, L., et al., *Comment on "Efficacy and safety of photodynamic therapy with amino-5-laevulinate nanoemulsion versus methyl-5-aminolaevulinate for actinic keratosis: A meta-analysis".* Photodiagnosis Photodyn Ther, 2020. 32: p. 101507.

15. Liew, Y.C.C., et al., *Photodynamic therapy for the prevention and treatment of actinic keratosis/squamous cell carcinoma in solid organ transplant recipients: a systematic review and meta-analysis.* J Eur Acad Dermatol Venereol, 2020. 34(2): p. 251-259.

16. Mpourazanis, G., et al., *The effectiveness of photodynamic therapy and cryotherapy on patients with basal cell carcinoma: A systematic review and meta-analysis.* Dermatol Ther, 2020. 33(6): p. e13881.

17. Snast, I., et al., *Nonsurgical Treatments for Extramammary Paget Disease: A Systematic Review and Meta-Analysis.* Dermatology, 2020. 236(6): p. 493-499.

18. Unanyan, A., et al., *Efficacy of photodynamic therapy in women with HSIL, LSIL and early stage squamous cervical cancer: a systematic review and meta-analysis.* Photodiagnosis Photodyn Ther, 2021. 36: p. 102530.

19. Binnal, A., et al., *Photodynamic therapy for oral potentially malignant disorders: A systematic review and meta-analysis.* Photodiagnosis Photodyn Ther, 2022. 37: p. 102713.

20. Choudhary, R., et al., *The Effect of Photodynamic Therapy on Oral-Premalignant Lesions: A Systematic Review.* J Clin Exp Dent, 2022. 14(3): p. e285-e292.

21. Ibarra, A.M.C., et al., *Photodynamic therapy for squamous cell carcinoma of the head and neck: narrative review focusing on photosensitizers.* Lasers Med Sci, 2022. 37(3): p. 1441-1470.

22. Song, S., et al., *Comparative efficacy and safety of local palliative therapeutics for unresectable malignant biliary obstruction: a Bayesian network meta-analysis.* Expert Rev Gastroenterol Hepatol, 2022. 16(6): p. 555-567.

23. Stewart, J.R., M.E. Lang, and J.D. Brewer, *Efficacy of nonexcisional treatment modalities for superficially invasive and in situ squamous cell carcinoma: A systematic review and meta-analysis.* J Am Acad Dermatol, 2022. 87(1): p. 131-137.

24. Yongpisarn, T., R. Rigo, and K. Minkis, *Durable Clearance Rate of Photodynamic Therapy for Bowen Disease and Cutaneous Squamous Cell Carcinoma: A Systematic Review and Meta-Analysis.* Dermatol Surg, 2022. 48(4): p. 395-400.

25. Fornel, D.G., et al., *Photodynamic Therapy Can Modulate the Nasopharyngeal Carcinoma Microenvironment Infected with the Epstein-Barr Virus: A Systematic Review and Meta-Analysis.* Biomedicines, 2023. 11(5).

26. Yuan, X., R. Xue, and X. Cao, *Network meta-analysis of treatments for perineal extramammary paget's disease: Focusing on performance of recurrence prevention.* PLoS One, 2023. 18(11): p. e0294152.

27. Shanazarov, N., et al., *Evaluation of the effectiveness and safety of photodynamic therapy in the treatment of precancerous diseases of the cervix (neoplasia) associated with the human papillomavirus: A systematic review.* Photodiagnosis Photodyn Ther, 2024. 45: p. 103925.
