# Supplementary material for: Umbrella review of photodynamic therapy for cancer: efficacy, safety, and clinical applications
Source: Front Oncol. 2025 Aug 4;15:1528314. doi: 10.3389/fonc.2025.1528314 (PMC12358287; doi:10.3389/fonc.2025.1528314)
Supplement: Supplementary Table 4 — The photosensitizers and parameters used for different cancers and representative studies. [file Table4.docx]

Table S4. The photosensitizers and parameters used for different cancers and representative studies.

| **Cancer** | **Photosensitizer** | **Route of Administration ^ƒ^** | **Dose ^†^** | **Wavelength** **^‡^** | **Light energy density** | **Power density ^£^** | **Main results ^§^** |
| --- | --- | --- | --- | --- | --- | --- | --- |
| Cholangiocarcinoma | Photosan-3 (1) | Intravenous injection | 2 mg/kg | 633 ± 3 nm | 180-200 J/cm^2^ | 450–500 mW/cm^2^ | Median survival time: 7  months for the control group; 21 months for the PDT group. |
|  | Photofrin (2) | Intravenous injection | 2 mg/kg | 633 ± 3 nm | 180-200 J/cm^2^ | 450–500 mW/cm^2^ | Median survival time: 98 days for the control group; 493 days for the PDT group. |
|  | Hematoporphyrin (3) | Intravenous injection | 5 mg/kg | 630 nm | 200-400 J/cm^2^ | 400 mW/cm^2^ | Median survival time: 9.8 months for the control group; 14.2 months for the PDT group. |
|  | Temoporfin (4) | Intravenous injection | 0.15 mg/kg | 652 nm | 20J/cm² | 100 mW/cm² | Median progression free survival: 96 days for the control group; 175 days for the PDT group. |
| Skin cancer | 5-Aminolevulinic acid (ALA) (5) | Topical application | Topical 20% | 630 nm | 50-150J/cm² | 100-300 mW/cm² | 76% of superficial BCC had complete response, with a median  session number 3.7. |
|  | Methyl aminolevulinate (MAL) (6) | Topical application | Topical 16% | 570-670 nm | 50-150J/cm² | 100-200 mW/cm² | There was no difference in 5-year recurrence rates with either treatment (20% with cryotherapy vs. 22% with MAL PDT). More patients had an excellent cosmetic outcome with MAL PDT (60% vs. 16% with cryotherapy). |
|  | Temoporfin (7) | Intravenous injection | 0.1-0.2 mg/kg | 652nm | 10-50J/cm² | 100-300 mW/cm² | Ninety tumours (92.7%) showed a complete response with an excellent cosmetic outcome. |
|  | Photosan-3 (8) | Intravenous injection | 2 mg/kg | 630 nm | 100 J/cm^2^ | 100 mW/cm^2^ | After the longest follow up of 14 months two patients revealed recurrence of the disease or residual tumor, two patients have been retreated because of residual dysplastic cells in the control biopsy, and all other patients stay histologically proven free of disease. |
| Prostate Cancer | Temoporfin (9) | Intravenous injection | 0.15 mg/kg | 652 nm | 50 J/cm² | 100 mW/cm² | Photodynamic therapy was given to 14 men using high light doses in 13. Treatment was well tolerated. PSA decreased in 9 patients (to undetectable levels in 2) and 5 had no viable tumor on posttreatment biopsies. |
|  | 5-Aminolevulinic acid (ALA) (10) | Orally | 20 mg/kg | 633 nm | 250 J/cm² | 100-300 mW/cm² | 6 weeks after interstitial PDT the PSA values were reduced by 20% up to 70%. Regarding the side effects no patient complained about incontinence or dysuria after PDT. |
|  | Photofrin (11) | Intravenous injection | 2.5 mg/kg | 638 nm | 15 J/cm^2^ | 100-300 mW/cm² | PSA decreased significantly after PDT treatment. |
|  | Motexafin lutetium (12) | Intravenous injection | 0.5-2 mg/kg | 732 nm | 25–150J/cm^2^ | 100 - 300 mW/cm² | PDT to induce large, transient increases in serum PSA levels. Patients who experienced high PDT dose demonstrated greater short-term increase in PSA and a significantly more durable PSA response (biochemical delay). |
|  | Padoporfin (13) | Intravenous injection | 0.10–2.00 mg/kg | 763 nm | 100-200J/cm² | 100-300 mW/cm² | TOOKAD-VTP can produce large avascular regions in the irradiated prostate, and result in a complete negative-biopsy response at high light doses. A response rate of more than half for those patients receiving the highest light doses. |
|  | Padeliporfin (14) | Intravenous injection | 2-6 mg/kg | 753 nm | 200J/cm^2^ | 150 mW/cm^2^ | Treatment with 4 mg/kg TOOKAD Soluble activated by 753 nm light at a dose of 200 J/cm and an LDI of >1 resulted in treatment effect in 95% of the planned treatment volume and a negative biopsy rate at 6 months of 10/12 men (83%). |
| Oral cancer | Talaporfin sodium (15) | Intravenous injection | 40 mg/m² | 664 nm | 50-150J/cm² | 100-300 mW/cm² | Complete response was achieved in six of eight cases, and two cases showed partial response as a clinical outcome of t-PDT. Recurrence occurred in one of the CR cases 9 months after irradiation. |
|  | Photofrin (16) | Intravenous injection | 2 mg/kg | 630 nm | 100-200 J/cm^2^ | 100-300 mW/cm^2^ | Six months after PDT, 30 patients (88.2%) showed complete responses while 9 patients (26.5%) had local relapses during long-term follow-up. The 5-year overall survival, disease-specific survival, and disease-free survival rates were 76.5%, 84.6%, and 63.3%, respectively. |
|  | Chlorin-based compound, 3-(1’-hexyloxyethyl) pyropheophorbide (HPPH) (17) | Intravenous injection | 4 mg/kg | 665 nm | 100-150J/cm² | 100 mW/cm² | Complete response rates were 46% for dysplasia and carcinoma in situ and 82% for squamous cell carcinomas lesions at 140 J/cm^2^. |
|  | Meta-tetra (hydroxyphenyl) chlorin (mTHPC) (18) | Intravenous injection | 0.05–0.15 mg/kg | 652 nm | 10–20 J/cm² | 10–100 mW/cm² | A complete tumour response was achieved in 85% of protocol-compliant patients (97 of 114 patients). A complete response was maintained in 85% of responders at 1 year and in 77% at 2 years. One- and 2-year actuarial survival rates were 89% and 75%, respectively. |

**^ƒ^** The administration route of photosensitizers.

**^†^** The dosage of the photosensitizer.

**^‡^** The wavelength of the light source.

**^£^** The power density of the light source.

**^§^** The main results in the original study.

**Reference**

1. Zoepf T, Jakobs R, Arnold JC, Apel D, Riemann JF. Palliation of nonresectable bile duct cancer: improved survival after photodynamic therapy. Am J Gastroenterol. 2005;100(11):2426-30.

2. Ortner ME, Caca K, Berr F, Liebetruth J, Mansmann U, Huster D, et al. Successful photodynamic therapy for nonresectable cholangiocarcinoma: a randomized prospective study. Gastroenterology. 2003;125(5):1355-63.

3. Li Z, Jiang X, Xiao H, Chen S, Zhu W, Lu H, et al. Long-term results of ERCP- or PTCS-directed photodynamic therapy for unresectable hilar cholangiocarcinoma. Surg Endosc. 2021;35(10):5655-64.

4. Hauge T, Hauge PW, Warloe T, Drolsum A, Johansen C, Viktil E, et al. Randomised controlled trial of temoporfin photodynamic therapy plus chemotherapy in nonresectable biliary carcinoma--PCS Nordic study. Photodiagnosis Photodyn Ther. 2016;13:330-3.

5. Baptista J, Martinez C, Leite L, Cochito M. Our PDT experience in the treatment of non-melanoma skin cancer over the last 7 years. J Eur Acad Dermatol Venereol. 2006;20(6):693-7.

6. Basset-Seguin N, Ibbotson SH, Emtestam L, Tarstedt M, Morton C, Maroti M, et al. Topical methyl aminolaevulinate photodynamic therapy versus cryotherapy for superficial basal cell carcinoma: a 5 year randomized trial. Eur J Dermatol. 2008;18(5):547-53.

7. Kubler AC, Haase T, Staff C, Kahle B, Rheinwald M, Muhling J. Photodynamic therapy of primary nonmelanomatous skin tumours of the head and neck. Lasers Surg Med. 1999;25(1):60-8.

8. Feyh J, Goetz A, Muller W, Konigsberger R, Kastenbauer E. Photodynamic therapy in head and neck surgery. J Photochem Photobiol B. 1990;7(2-4):353-8.

9. Nathan TR, Whitelaw DE, Chang SC, Lees WR, Ripley PM, Payne H, et al. Photodynamic therapy for prostate cancer recurrence after radiotherapy: a phase I study. J Urol. 2002;168(4 Pt 1):1427-32.

10. Zaak D, Sroka R, Höppner M, Khoder W, Reich O, Tritschler S, et al. Photodynamic Therapy by Means of 5-ALA Induced PPIX in Human Prostate Cancer – Preliminary Results. Medical Laser Application. 2003;18(1):91-5.

11. Windahl T, Andersson SO, Lofgren L. Photodynamic therapy of localised prostatic cancer. Lancet. 1990;336(8723):1139.

12. Patel H, Mick R, Finlay J, Zhu TC, Rickter E, Cengel KA, et al. Motexafin lutetium-photodynamic therapy of prostate cancer: short- and long-term effects on prostate-specific antigen. Clin Cancer Res. 2008;14(15):4869-76.

13. Trachtenberg J, Weersink RA, Davidson SR, Haider MA, Bogaards A, Gertner MR, et al. Vascular-targeted photodynamic therapy (padoporfin, WST09) for recurrent prostate cancer after failure of external beam radiotherapy: a study of escalating light doses. BJU Int. 2008;102(5):556-62.

14. Moore CM, Azzouzi AR, Barret E, Villers A, Muir GH, Barber NJ, et al. Determination of optimal drug dose and light dose index to achieve minimally invasive focal ablation of localised prostate cancer using WST11-vascular-targeted photodynamic (VTP) therapy. BJU Int. 2015;116(6):888-96.

15. Ikeda H, Ohba S, Egashira K, Asahina I. The effect of photodynamic therapy with talaporfin sodium, a second-generation photosensitizer, on oral squamous cell carcinoma: A series of eight cases. Photodiagnosis Photodyn Ther. 2018;21:176-80.

16. Toratani S, Tani R, Kanda T, Koizumi K, Yoshioka Y, Okamoto T. Photodynamic therapy using Photofrin and excimer dye laser treatment for superficial oral squamous cell carcinomas with long-term follow up. Photodiagnosis Photodyn Ther. 2016;14:104-10.

17. Rigual N, Shafirstein G, Cooper MT, Baumann H, Bellnier DA, Sunar U, et al. Photodynamic therapy with 3-(1'-hexyloxyethyl) pyropheophorbide a for cancer of the oral cavity. Clin Cancer Res. 2013;19(23):6605-13.

18. Hopper C, Kubler A, Lewis H, Tan IB, Putnam G. mTHPC-mediated photodynamic therapy for early oral squamous cell carcinoma. Int J Cancer. 2004;111(1):138-46.
