# Supplementary material for: Umbrella review of photodynamic therapy for cancer: efficacy, safety, and clinical applications
Source: Front Oncol. 2025 Aug 4;15:1528314. doi: 10.3389/fonc.2025.1528314 (PMC12358287; doi:10.3389/fonc.2025.1528314)
Supplement: Supplementary Table 5 — Detailed results of included and excluded associations. [file Table5.docx]

Table S5. Detailed results of included and excluded associations.

| **Included associations** | | | | | | | | | | | | | | | | | | | | | | |
| --- | --- | --- | --- | --- | --- | --- | --- | --- | --- | --- | --- | --- | --- | --- | --- | --- | --- | --- | --- | --- | --- | --- |
| **Author** | **Cancer** | **Intervention** | **Comparison** | **Outcome** | **No. studies ^ƒ^** | **No. of patients^†^** | **Metric** | **Reported MA model** | **Reported p-value** | **Reported ES (95%CI)** | **Reported I^2^** | **ES (95%CI) of largest study^‡^** | **Consistence of individual study (Y/N) ^£^** | **RA model** | **RA ES (95%CI)** | **P value ^§^** | **PI ES (95%)** | **RA I^2^** | **Egger p-value****^¶^** | **TES p-value****^€^** | **Class** | **AMSTAR 2** |
| Leggett 2012 | Unresectable cholangiocarcinoma | Biliary stenting with PDT | Biliary stenting | Mortality | 6 | 170/157 | RR | NA | 0.050 | 0.90 (0.80, 1.00) | NA | 1.02 (0.92, 1.13) | N | DL | 0.630 (0.478, 0.830) | 0.001 | (0.340, 1.168) | 24.886 | 0.788 | 0.840 | IV | CL |
| Leggett 2012 | Unresectable cholangiocarcinoma | Biliary stenting with PDT | Biliary stenting | Length of survival | 6 | 170/157 | WMD | NA | <0.001 | 264.88 (153.80, 375.95) | NA | 168.00 (45.69, 290.31) | N | DL | 250.749 (150.631, 350.866) | <0.001 | (0, Inf) | 51.417 | 0.207 | 0.015 | IV | CL |
| Leggett 2012 | Unresectable cholangiocarcinoma | Biliary stenting with PDT | Biliary stenting | Karnofsky scores | 3 | 104/91 | WMD | NA | <0.001 | 7.74 (3.73, 11.76) | NA | 11.00 (5.30, 16.70) | N | HKSJ | 7.578 (-0.983, 16.139) | 0.063 | (0, 2.373e+30) | 14.535 | 0.900 | 0.840 | ns | CL |
| Leggett 2012 | Unresectable cholangiocarcinoma | Biliary stenting with PDT | Biliary stenting | Bilirubin | 5 | 147/140 | WMD | NA | 0.220 | -2.92 (-7.54, -1.71) | NA | -3.60 (-6.83, -0.37) | N | DL | -2.840 (-7.183, 1.503) | 0.200 | (0, 3.260e+10) | 91.709 | 0.157 | <0.001 | ns | CL |
| Lu 2015 | Unresectable cholangiocarcinoma | Biliary stenting with PDT | Biliary stenting | OS | 7 | 226/336 | HR | Random | 0.0005 | 0.49 (0.33, 0.73) | 70 (0.003) | 0.79 (0.51, 1.22) | N | DL | 0.488 (0.327, 0.729) | <0.001 | (0.146, 1.633) | 69.797 | 0.234 | 0.205 | IV | CL |
| Chen 2022 | Hilar cholangiocarcinoma | Biliary stenting with PDT | Biliary stenting | OS | 2 | 205 | HR | Random | 0.002 | 0.58 (0.40, 0.82) | 19 (0.266) | 0.65 (0.45, 0.94) | Y | HKSJ | 0.573 (0.057, 5.732) | 0.200 | NA | 17.783 | NA | NA | ns | CL |
| Yu 2023 | Unresectable extrahepatic cholangiocarcinoma | PDT with chemotherapy | Chemotherapy alone | OS | 3 | 261 | HR | Fixed | 0.020 | 0.69 (0.51, 0.94) | 0 (0.910) | 0.70 (0.51, 0.96) | N | HKSJ | 0.690 (0.566, 0.842) | 0.015 | (0.384, 1.241) | 0 | 0.026 | 0.378 | IV | L |
| Yu 2023 | Unresectable extrahepatic cholangiocarcinoma | PDT with chemotherapy | PDT alone | OS | 3 | 185 | HR | Fixed | <0.01 | 0.36 (0.23, 0.56) | 0 (0.440) | 0.45 (0.24, 0.84) | Y | HKSJ | 0.363 (0.152, 0.870) | 0.038 | (0.028, 4.784) | 0 | 0.004 | 0.024 | IV | L |
| Yu 2023 | Unresectable extrahepatic cholangiocarcinoma | PDT with chemotherapy | Chemotherapy alone | AEs (cholangitis) | 2 | 43/34 | OR | Fixed | 0.110 | 2.11 (0.84, 5.31) | 21 (0.260) | 2.81 (0.97, 8.13) | Y | HKSJ | 1.958 (0.001, 3502.183) | 0.458 | NA | 21.216 | NA | NA | ns | L |
| Yu 2023 | Unresectable extrahepatic cholangiocarcinoma | PDT with chemotherapy | Chemotherapy alone | AEs (abscess) | 2 | 43/34 | OR | Fixed | 0.180 | 2.74 (0.62, 12.18) | 0 (0.460) | 1.94 (0.35, 10.85) | Y | HKSJ | 2.635 (0.002, 3979.188) | 0.342 | NA | 0 | NA | NA | ns | L |
| Yu 2023 | Unresectable extrahepatic cholangiocarcinoma | PDT with chemotherapy | Chemotherapy alone | AEs (photosensitivity reaction) | 1 | 36/26 | OR | Fixed | NA | 7.34 (0.38, 142.53) | NA | 7.34 (0.38, 142.53) | NA | HKSJ | 7.338 (0.378, 142.532) | 0.188 | NA | NA | NA | NA | ns | L |
| Yu 2023 | Unresectable extrahepatic cholangiocarcinoma | PDT with chemotherapy | PDT alone | AEs (cholangitis) | 3 | 85/127 | OR | Fixed | 0.500 | 1.26 (0.64, 2.51) | 0 (0.770) | 1.22 (0.12, 12.61) | Y | HKSJ | 1.263 (0.586, 2.726) | 0.321 | (0.13, 12.238) | 0 | 0.834 | 0.664 | ns | L |
| Yu 2023 | Unresectable extrahepatic cholangiocarcinoma | PDT with chemotherapy | PDT alone | AEs (abscess) | 3 | 73/114 | OR | Fixed | 0.270 | 2.05 (0.58, 7.28) | 36 (0.210) | 0.68 (0.03, 14.98) | Y | HKSJ | 1.490 (0.020, 112.328) | 0.730 | (0, 8.598e+7) | 31.839 | 0.697 | 0.695 | ns | L |
| Yu 2023 | Unresectable extrahepatic cholangiocarcinoma | PDT with chemotherapy | PDT alone | AEs (photosensitivity reaction) | 3 | 73/114 | OR | Fixed | 0.870 | 1.10 (0.35, 3.44) | 0 (0.940) | 0.68 (0.03, 14.98) | Y | HKSJ | 1.107 (0.575, 2.129) | 0.574 | (0.16, 7.643) | 0 | 0.003 | 0.622 | ns | L |
| Wang 2015 | BCC | PDT | Surgery | 1 year recurrence | 3 | 244/254 | RR | Random | 0.003 | 12.42 (2.34, 66.02) | 0 (0.770) | 22.81 (1.36, 382.61) | N | HKSJ | 1.996 (1.791, 2.225) | 0.001 | (1.449, 2.749) | 0 | 0.389 | 0.280 | IV | CL |
| Zou 2016 | BCC | PDT | Surgery | 1 year recurrence | 4 | 210/211 | RR | Fixed | 0.002 | 5.28 (1.85, 15.12) | 0 (0.830) | 5.83 (1.33, 25.53) | N | HKSJ | 1.822 (1.521, 2.183) | 0.002 | (1.427, 2.327) | 0 | 0.250 | 0.753 | IV | L |
| Wang 2020 | BCC | MAL-PDT | Surgery, ALA-PDT, cryotherapy, imiquimod, fluorouracil | 1 year recurrence | 6 | 773/783 | RR | Random | 0.100 | 1.43 (0.93, 2.19) | 49 (0.080) | 1.35 (0.93, 1.94) | N | DL | 1.376 (1.071, 1.767) | 0.012 | (0.605, 3.127) | 79.539 | 0.341 | 0.693 | IV | CL |
| Zou 2016 | BCC | PDT | Surgery | 2-year recurrence | 3 | 189/192 | RR | Fixed | 0.0002 | 6.48 (2.46, 17.09) | 0 (0.400) | 9.54 (2.28, 39.86) | N | HKSJ | 1.946 (1.376, 2.753) | 0.014 | (0.699, 5.42) | 0 | 0.037 | 0.917 | IV | L |
| Zou 2016 | BCC | PDT | Surgery | 3-year recurrence | 2 | 136/140 | RR | Fixed | 0.0001 | 9.67 (3.02, 30.99) | 0 (0.700) | 11.13 (2.69, 46.01) | Y | HKSJ | 2.068 (0.773, 5.530) | 0.068 | NA | 0 | NA | NA | ns | L |
| Zou 2016 | BCC | PDT | Surgery | 4-year recurrence | 2 | 136/140 | RR | Fixed | <0.001 | 7.73 (2.81, 21.28) | 33 (0.220) | 12.19 (2.97, 50.11) | N | HKSJ | 1.997 (0.282, 14.116) | 0.139 | NA | 45.465 | NA | NA | ns | L |
| Wang 2020 | BCC | MAL-PDT | Surgery, cryotherapy, imiquimod, fluorouracil | 5-year recurrence | 4 | 541/532 | RR | Random | 0.030 | 1.45 (1.05, 2.02) | 49 (0.120) | 1.24 (0.93, 1.66) | N | HKSJ | 1.301 (0.955, 1.771) | 0.073 | (0.648, 2.611) | 49.624 | 0.960 | 0.499 | ns | CL |
| Wang 2020 | BCC | MAL-PDT | Surgery, ALA-PDT, Placebo, cryotherapy, Imiquimod | Cosmetic outcome | 8 | 729/709 | RR | Random | 0.110 | 1.15 (0.97, 1.36) | 86 (<0.0001) | 1.08 (0.92, 1.28) | N | DL | 1.300 (0.941, 1.797) | 0.112 | (0.469, 3.606) | 80.153 | 0.184 | 0.389 | ns | CL |
| Wang 2015 | BCC | PDT | Surgery, cryotherapy, topical therapy, placebo | Complete clearance | 7 | 667/833 | RR | Random | 0.470 | 0.97 (0.88, 1.06) | 86 (<0.0001) | 0.95 (0.88, 1.02) | N | DL | 0.770 (0.524, 1.133) | 0.185 | (0.201, 2.955) | 88.489 | 0.533 | 0.008 | ns | CL |
| Wang 2020 | BCC | MAL-PDT | Surgery, ALA-PDT, placebo, cryotherapy, imiquimod | 3 months complete response | 8 | 957/957 | RR | Random | 0.690 | 0.99 (0.92, 1.05) | 83 (<0.0001) | 0.96 (0.88, 1.04) | N | DL | 0.915 (0.647, 1.295) | 0.618 | (0.283, 2.958) | 86.329 | 0.333 | 0.432 | ns | CL |
| Collier 2018 | BCC | PDT | Surgery | 3 months initial clearance | 2 | 138/140 | RR | Fixed | 0.030 | 0.94 (0.89, 0.99) | 0 (0.620) | 0.95 (0.89, 1.02) | Y | HKSJ | 0.608 (0.338, 1.096) | 0.059 | NA | 0 | NA | NA | ns | L |
| Wang 2020 | BCC | MAL-PDT | Surgery, ALA-PDT, placebo, cryotherapy, imiquimod | 1-year complete response | 6 | 693/730 | RR | Random | 0.020 | 0.95 (0.91, 0.99) | NA | 0.95 (0.88, 1.03) | N | DL | 0.720 (0.558, 0.930) | 0.012 | (0.317, 1.637) | 73.527 | 0.028 | 0.739 | IV | CL |
| Zou 2016 | BCC | PDT | Surgery | 2-year complete response | 3 | 219/228 | RR | Random | 0.040 | 0.83 (0.69, 1.00) | 61 (0.080) | 0.77 (0.65, 0.91) | N | HKSJ | 0.676 (0.440, 1.039) | 0.059 | (0.181, 2.522) | 2.428 | 0.273 | 0.416 | ns | L |
| Zou 2016 | BCC | PDT | Surgery | 3-year complete response | 3 | 219/228 | RR | Fixed | <0.0001 | 0.73 (0.63, 0.85) | 28 (0.250) | 0.74 (0.60, 0.90) | N | HKSJ | 0.645 (0.532, 0.782) | 0.010 | (0.366, 1.137) | 0 | 0.268 | 0.565 | IV | L |
| Zou 2016 | BCC | PDT | Surgery | 4-year complete response | 2 | 136/140 | RR | Fixed | 0.180 | 0.84 (0.65, 1.08) | 0 (0.570) | 0.73 (0.36, 1.48) | Y | HKSJ | 0.811 (0.770, 0.854) | 0.012 | NA | 0 | NA | NA | IV | L |
| Zou 2016 | BCC | PDT | Surgery | 5-year complete response | 2 | 136/140 | RR | Random | 0.090 | 0.79 (0.61, 1.03) | 56 (0.130) | 0.70 (0.54, 0.89) | N | HKSJ | 0.710 (0.110, 4.588) | 0.257 | NA | 29.163 | NA | NA | ns | L |
| Gu 2021 | BCC | Laser-assisted PDT | Conventional PDT | Complete response rate | 2 | 37/37 | RR | Random | 0.310 | 1.94 (0.55, 6.93) | 82 (0.020) | 3.75 (1.49, 9.43) | N | HKSJ | 1.858 (0.004, 915.962) | 0.425 | NA | 71.201 | NA | NA | ns | L |
| Collier 2018 | BCC | PDT | Surgery | 1-year sustained clearance | 3 | 148/156 | RR | Fixed | 0.006 | 0.90 (0.84, 0.97) | 0 (0.600) | 0.90 (0.82, 0.99) | N | HKSJ | 0.609 (0.496, 0.747) | 0.009 | (0.332, 1.115) | 0 | 0.494 | 0.371 | IV | L |
| Wang 2020 | BCC | MAL-PDT | Surgery, ALA-PDT, placebo, cryotherapy, imiquimod | AEs | 5 | 421/406 | RR | Random | 0.490 | 1.41 (0.53, 3.76) | 99 (<0.0001) | 1.00 (0.99, 1.01) | N | DL | 1.467 (1.022, 2.107) | 0.038 | (0.468, 4.598) | 65.637 | 0.980 | 0.055 | IV | CL |
| Ou-yang 2023 | BCC | MAL-PDT | Placebo | Pain | 1 | 66/65 | RR | Random | 0.030 | 3.94 (1.17, 13.22) | NA | 3.94 (1.17, 13.22) | NA | HKSJ | 1.719 (1.249, 2.365) | 0.001 | NA | only 1 study | NA | NA | IV | L |
| Ou-yang 2023 | BCC | MAL-PDT | Surgery | Pain | 2 | 152/145 | RR | Random | 0.870 | 1.13 (0.26, 4.97) | NA | 0.48 (0.09, 2.56) | Y | HKSJ | 1.146 (0.014, 95.001) | 0.761 | NA | 36.178 | NA | NA | ns | L |
| Ou-yang 2023 | BCC | MAL-PDT | Fluorouracil | Pain | 1 | 190/192 | RR | Random | 0.030 | 1.95 (1.06, 3.60) | NA | 1.95 (1.06, 3.60) | NA | HKSJ | 1.378 (1.076, 1.763) | 0.011 | NA | only 1 study | NA | NA | IV | L |
| Ou-yang 2023 | BCC | MAL-PDT | Imiquimod | Pain | 1 | 190/189 | RR | Random | 0.003 | 2.98 (1.44, 6.17) | NA | 2.98 (1.44, 6.17) | NA | HKSJ | 1.578 (1.268, 1.965) | <0.001 | NA | only 1 study | NA | NA | IV | L |
| Ou-yang 2023 | BCC | MAL-PDT | ALA-PDT | Pain | 1 | 143/138 | RR | Random | 0.070 | 1.03 (1.00, 1.06) | NA | 1.03 (1.00, 1.06) | NA | HKSJ | 5.162 (0.371, 71.760) | 0.222 | NA | only 1 study | NA | NA | ns | L |
| Ou-yang 2023 | SCC | ALA-PDT | Cryotherapy | Response at 3 months | 1 | 20/20 | RR | Random | 1 | 1.00 (0.91, 1.10) | NA | 1.00 (0.91, 1.10) | NA | HKSJ | 1 (0.138, 7.270) | 1.000 | NA | only 1 study | NA | NA | ns | L |
| Ou-yang 2023 | SCC | ALA-PDT | Fluorouracil | Response at 3 months | 1 | 33/33 | RR | Random | 0.050 | 1.32 (1.00, 1.73) | NA | 1.32 (1.00, 1.73) | NA | HKSJ | 2.132 (0.891, 5.103) | 0.089 | NA | only 1 study | NA | NA | ns | L |
| Gu 2021 | SCC | Laser-assisted PDT | Conventional PDT | Complete response rate | 3 | 102/91 | RR | Random | <0.001 | 1.85 (1.29, 2.67) | 46 (0.150) | 1.57 (1.16, 2.14) | Y | HKSJ | 2.748 (2.187, 3.452) | 0.003 | (1.401, 5.390) | 0 | 0.777 | 0.086 | IV | L |
| Ou-yang 2023 | SCC | MAL-PDT | Placebo | Recurrence at 12 months | 1 | 103/4 | RR | Random | 0.030 | 0.29 (0.10, 0.86) | NA | 0.29 (0.10, 0.86) | NA | HKSJ | 0.902 (0.757, 1.076) | 0.254 | NA | only 1 study | NA | NA | ns | L |
| Ou-yang 2023 | SCC | MAL-PDT | Cryotherapy | Recurrence at 12 months | 1 | 103/73 | RR | Random | 0.300 | 0.71 (0.37, 1.36) | NA | 0.71 (0.37, 1.36) | NA | HKSJ | 0.83 (0.567, 1.215) | 0.337 | NA | only 1 study | NA | NA | ns | L |
| Ou-yang 2023 | SCC | ALA-PDT | Fluorouracil | Recurrence at 12 months | 1 | 33/33 | RR | Random | 0.160 | 0.33 (0.07, 1.53) |  | 0.33 (0.07, 1.53) | NA | HKSJ | 0.468 (0.138, 1.591) | 0.224 | NA | only 1 study | NA | NA | ns | L |
| Ou-yang 2023 | SCC | MAL-PDT | YAG-AFL-PDT | Recurrence at 24 months | 1 | 19/21 | RR | Random | 0.004 | 3.87 (1.54, 9.72) | NA | 3.87 (1.54, 9.72) | NA | HKSJ | 3.422 (1.524, 7.686) | 0.003 | NA | only 1 study | NA | NA | IV | L |
| Ou-yang 2023 | SCC | MAL-PDT | Cryotherapy | Cosmetic rating good at 12 months | 1 | 82/65 | RR | Random | 0.0002 | 1.42 (1.18, 1.70) | NA | 1.42 (1.18, 1.70) | NA | HKSJ | 3.465 (1.553, 7.73) | 0.002 | NA | only 1 study | NA | NA | IV | L |
| Ou-yang 2023 | SCC | MAL-PDT | Fluorouracil | Cosmetic rating good at 12 months | 1 | 82/21 | RR | Random | 0.090 | 1.23 (0.96, 1.58) | NA | 1.23 (0.96, 1.58) | NA | HKSJ | 1.656 (0.885, 3.099) | 0.115 | NA | only 1 study | NA | NA | ns | L |
| Ou-yang 2023 | SCC | MAL-PDT | Cryotherapy | AEs | 1 | 96/82 | RR | Random | 0.070 | 1.28 (0.98, 1.68) | NA | 1.28 (0.98, 1.68) | NA | HKSJ | 1.3 (0.974, 1.734) | 0.074 | NA | only 1 study | NA | NA | ns | L |
| Ou-yang 2023 | SCC | MAL-PDT | Fluorouracil | AEs | 1 | 96/30 | RR | Random | 0.110 | 0.82 (0.63, 1.05) | NA | 0.82 (0.63, 1.05) | NA | HKSJ | 0.863 (0.716, 1.041) | 0.125 | NA | only 1 study | NA | NA | ns | L |
| Ou-yang 2023 | SCC | ALA-PDT | Cryotherapy | Pain | 1 | 20/20 | RR | Random | 0.009 | 0.58 (0.38, 0.87) | NA | 0.58 (0.38, 0.87) | NA | HKSJ | 0.407 (0.244, 0.681) | 0.001 | NA | only 1 study | NA | NA | IV | L |
| Ou-yang 2023 | SCC | ALA-PDT | Fluorouracil | Pain | 1 | 19/15 | RR | Random | 0.660 | 1.11 (0.71, 1.73) | NA | 1.11 (0.71, 1.73) | NA | HKSJ | 1.167 (0.576, 2.364) | 0.669 | NA | only 1 study | NA | NA | ns | L |
| Gu 2021 | SCC | laser-assisted PDT | Conventional PDT | Complete response rate | 3 | 102/91 | RR | Random | <0.001 | 1.85 (1.29, 2.67) | 46 (0.150) | 1.57 (1.16, 2.14) | Y | HKSJ | 2.748 (2.187, 3.452) | 0.003 | (1.401, 5.39) | 0 | 0.777 | 0.086 | IV | L |
| Ou-yang 2023 | BCC and SCC | MAL-PDT | Placebo | Response at 3 months | 2 | 186/94 | RR | Random | <0.0001 | 3.00 (2.05, 4.39) | 4 (0.310) | 4.41 (1.84, 10.54) | Y | HKSJ | 2.756 (2.653, 2.863) | 0.002 | NA | 0 | NA | NA | IV | L |
| Ou-yang 2023 | BCC and SCC | MAL-PDT | Fluorouracil | Response at 3 months | 2 | 307/227 | RR | Random | 0.830 | 1.02 (0.88, 1.18) | 62 (0.110) | 0.96 (0.88, 1.04) | Y | HKSJ | 1.026 (0.073, 14.388) | 0.921 | NA | 62.457 | NA | NA | ns | L |
| Ou-yang 2023 | BCC and SCC | MAL-PDT | Cryotherapy | Response at 3 months | 2 | 177/143 | RR | Random | 0.400 | 1.63 (0.53, 4.99) | 96 (<0.00001) | 1.08 (0.98, 1.19) | N | HKSJ | 1.916 (0.114, 32.144) | 0.209 | NA | 42.09 | NA | NA | ns | L |
| Ou-yang 2023 | BCC and SCC | MAL-PDT | YAG-AFL-PDT | Response at 3 months | 3 | 57/60 | RR | Random | 0.004 | 0.69 (0.53, 0.89) | 0 (0.580) | 0.62 (0.37, 1.06) | Y | HKSJ | 0.544 (0.460, 0.642) | 0.004 | (0.333, 0.889) | 0 | 0.189 | 0.708 | IV | L |
| Ou-yang 2023 | BCC and SCC | MAL-PDT | YAG-AFL-PDT | Response at 12 months | 3 | 57/60 | RR | Random | 0.002 | 0.39 (0.22, 0.71) | NA | 0.56 (0.34, 0.94) | Y | HKSJ | 0.378 (0.175, 0.818) | 0.032 | (0.039, 3.692) | 0 | 0.004 | 0.005 | IV | L |
| Ou-yang 2023 | BCC and SCC | MAL-PDT | Fluorouracil | Recurrence at 12 months | 2 | 259/193 | RR | Random | 0.740 | 1.11 (0.59, 2.09) | 0 (0.550) | 1.30 (0.58, 2.92) | N | HKSJ | 1.016 (0.387, 2.670) | 0.869 | NA | 0 | NA | NA | ns | L |
| Ou-yang 2023 | BCC and SCC | MAL-PDT | YAG-AFL-PDT | Recurrence at 12 months | 3 | 56/59 | RR | Random | <0.0001 | 5.66 (2.38, 13.46) | NA | 4.42 (1.47, 13.31) | N | HKSJ | 2.559 (1.466, 4.468) | 0.019 | (0.494, 13.266) | 0 | 0.401 | 0.231 | IV | L |
| Ou-yang 2023 | BCC and SCC | MAL-PDT | YAG-AFL-PDT | Cosmetic rating good at 12 months | 2 | 37/38 | RR | Random | 0.360 | 1.05 (0.94, 1.18) | 0 (0.920) | 1.05 (0.91, 1.21) | Y | HKSJ | 1.675 (0.367, 7.654) | 0.145 | NA | 0 | NA | NA | ns | L |
| Ou-yang 2023 | BCC and SCC | MAL-PDT | ALA-PDT | Cosmetic rating good at 12 months | 2 | 129/129 | RR | Random | 0.100 | 0.88 (0.75, 1.02) | 0 (0.500) | 0.84 (0.69, 1.03) | Y | HKSJ | 0.798 (0.236, 2.697) | 0.256 | NA | 0 | NA | NA | ns | L |
| Ou-yang 2023 | BCC and SCC | MAL-PDT | Placebo | AEs | 2 | 162/82 | RR | Random | 0.020 | 1.30 (1.04, 1.61) | 19 (0.020) | 1.06 (0.69, 1.63) | N | HKSJ | 1.56 (0.003, 727.7) | 0.527 | NA | 84.814 | NA | NA | ns | L |
| Ou-yang 2023 | BCC and SCC | MAL-PDT | YAG-AFL-PDT | AEs | 3 | 55/55 | RR | Random | 1 | 1.00 (0.94, 1.06) | NA | 1.00 (0.91, 1.10) | Y | HKSJ | 1.002 (0.879, 1.142) | 0.957 | (0.680, 1.476) | 0 | 0.364 | 0.609 | ns | L |
| Gu 2021 | BCC and SCC | Laser-assisted PDT | Conventional PDT | Complete response rate | 5 | 139/128 | RR | Random | 0.001 | 1.85 (1.26, 2.68) | 56 (0.060) | 1.57 (1.16, 2.14) | N | DL | 2.349 (1.639, 3.368) | <0.001 | (1.037, 5.322) | 19.059 | 0.895 | 0.096 | IV | L |
| Gu 2021 | BCC and SCC | Laser-assisted PDT | Conventional PDT | Complete response rate | 5 | 139/128 | RR | Random | 0.001 | 1.85 (1.26, 2.68) | 56 (0.060) | 1.57 (1.16, 2.14) | N | DL | 2.349 (1.639, 3.368) | <0.001 | (1.037, 5.322) | 19.059 | 0.895 | 0.096 | IV | L |
| Zhong 2020 | Bowen’s disease | PDT | 5-FU, cryotherapy | Lesion reduction | 6 | 320/199 | OR | Fixed | <0.00001 | 2.86 (1.89, 4.33) | 41 (0.130) | 2.08 (1.07, 4.03) | N | DL | 3.193 (1.230, 8.288) | 0.026 | (0.593, 17.187) | 40.721 | 0.005 | 0.006 | IV | L |
| Zhong 2020 | Bowen’s disease | PDT | 5-FU, cryotherapy | Recurrence rate | 3 | 281/142 | OR | Fixed | 0.080 | 0.59 (0.33, 1.07) | 0 (0.400) | 0.53 (0.24, 1.16) | N | HKSJ | 0.598 (0.167, 2.145) | 0.225 | (0.014, 26.024) | 0 | 0.897 | 0.773 | ns | L |
| Xue 2021 | Bowen's Disease | PDT | 5-FU, cryotherapy | Complete response rate (all) | 4 | 106/106 | RR | Random | 0.250 | 1.28 (0.84, 1.97) | 39 (0.180) | 0.70 (0.30, 1.62) | N | HKSJ | 1.243 (0.587, 2.629) | 0.424 | (0.206, 7.490) | 51.159 | 0.723 | 0.383 | ns | CL |
| **Excluded associations due to overlap** | | | | | | | | | | | | | | | | | | | | | | |
| Wang 2020 | BCC | MAL-PDT | Surgery | 1-year recurrence | 2 | 171/169 | RR | Random | 0.006 | 10.43 (1.98-55.03) | 0 (0.490) | 22.81 (1.36-382.61) | N | HKSJ | 1.969 (1.264, 3.065) | 0.033 | NA | 0 | NA | NA | IV | CL |
| Wang 2015 | BCC | PDT | Surgery, cryotherapy, topical therapy, placebo | 1-year recurrence | 6 | 544/720 | RR | Random | 0.11 | 1.85 (0.88-3.92) | 55 (0.050) | 1.67 (0.97-2.85) | N | DL | 1.566 (1.226, 2.000) | <0.0001 | (0.739, 3.320) | 67.238 | 6.76E-02 | 0.599 | IV | CL |
| Wang 2020 | BCC | MAL-PDT | Surgery | 5-year recurrence | 1 | 49/52 | RR | Random | NA | 3.71 (0.81-17.02) | NA | 3.71 (0.81-17.02) | NA | HKSJ | 1.704 (1.126, 2.578) | 0.012 | NA | NA | NA | NA | IV | CL |
| Wang 2015 | BCC | PDT | Cryotherapy | 5-year recurrence | 1 | 100/93 | RR | Random | 0.790 | 1.08 (0.62-1.86) | NA | 1.08 (0.62-1.86) | NA | HKSJ | 1.046 (0.756, 1.446) | 0.787 | NA | NA | NA | NA | ns | CL |
| Wang 2015 | BCC | PDT | Surgery, cryotherapy, topical therapy, placebo | 5-year recurrence | 3 | 226/230 | RR | Random | 0.150 | 3.06 (0.67-13.96) | 81 (0.005) | 1.08 (0.62-1.86) | N | HKSJ | 1.577 (0.620, 4.015) | 0.171 | (0.007, 374.521) | 83.833 | 0.656 | 0.513 | ns | CL |
| Wang 2015 | BCC | PDT | Surgery | Cosmetic outcome | 2 | 125/131 | RR | Random | <0.0001 | 1.87 (1.54-2.26) | 0 (0.550) | 1.81 (1.46-2.25) | Y | HKSJ | 3.554 (0.068, 185.98) | 0.153 | NA | 38.144 | NA | NA | ns | CL |
| Wang 2020 | BCC | MAL-PDT | Cryotherapy | Cosmetic outcome | 1 | 58/57 | RR | Random | NA | 1.05 (0.89-1.22) | NA | 1.05 (0.89-1.22) | NA | HKSJ | 1.160 (0.668, 2.013) | 0.598 | NA | NA | NA | NA | ns | CL |
| Wang 2015 | BCC | PDT | Surgery, cryotherapy, topical therapy, placebo | Cosmetic outcome | 5 | 453/638 | RR | Random | 0.001 | 1.54 (1.19-1.99) | 85 (<0.0001) | 1.54 (1.19-1.99) | N | DL | 2.613 (1.349, 5.061) | 0.004 | (0.236, 28.930) | 85.820 | 0.008 | <0.001 | IV | CL |
| Collier 2018 | BCC | PDT | Surgery, cryotherapy, topical therapy, placebo | Cosmetic outcome | 2 | 109/99 | RR | Fixed | <0.0001 | 3.73 (1.96-7.07) | 30 (0.230) | 2.67 (1.22-5.85) | Y | HKSJ | 1.870 (0.332, 10.544) | 0.136 | NA | 26.152 | NA | NA | ns | L |
| Zou 2016 | BCC | PDT | Surgery | Complete response | 2 | 136/140 | RR | Fixed | 0.040 | 0.95 (0.90-1.00) | 0 (0.480) | 0.96 (0.90-1.02) | Y | HKSJ | 0.615 (0.265, 1.428) | 0.086 | NA | 0 | NA | NA | ns | L |

**^ƒ^** In the original meta-analysis, the number of included studies on specific intervention measures and outcomes.

**^†^** In the original meta-analysis, the number of patients (intervention/comparison) on specific intervention measures and outcomes.

**^‡^** In the original meta-analysis, the effect size of the clinical study with the largest sample size.

**^£^**The original meta-analysis should assess whether the statistical significance of the effect size was consistent across individual clinical studies.

**^§^** The p-value resynthesized from the original meta-analysis.

**^¶^** The p-value of the egger test for the meta-analysis, which reflects publication bias or selective reporting bias.

**^€^** This is the P-value of the excess significance test for the meta-analysis, which reflects publication bias or selective reporting bias.

Abbreviation: AEs, Adverse events; ALA, 5-Aminolevulinic acid; AMSTAR 2, assessment of multiple systematic reviews; BCC, basal cell carcinoma; CL, critical low; CI, confidence interval; DL, DerSimonian-Laird; ES, effect size; HKSJ, Hartung-Knapp-Sidik-Jonkman; HR, hazard ratio; L, low; MA, meat-analysis; MAL, methyl aminolevulinate; N, no; NA, not available; OR, odds ratio; OS, overall survival; PDT, photodynamic therapy; PI, prediction interval; RA, re-analyze; RR, risk ratio; SCC, squamous cell carcinoma; TES, test of excess significance; WMD, weighted mean difference; Y, yes; YAG-AFL, erbium: yttrium-aluminum-garnet ablative factional laser; 5-FU, 5-Fluorouracil; IV, weak evidence (class IV); ns, non-significant (class ns).
