# Supplementary material for: Umbrella review of photodynamic therapy for cancer: efficacy, safety, and clinical applications
Source: Front Oncol. 2025 Aug 4;15:1528314. doi: 10.3389/fonc.2025.1528314 (PMC12358287; doi:10.3389/fonc.2025.1528314)
Supplement: Supplementary Table 6 — Detailed results of the association in single arm meta-analyses. [file Table6.docx]

Table S6. Detailed results of the association in single arm meta-analyses.

| **Author** | **Cancer** | **Intervention** | **Outcome** | **No. of studies ^ƒ^** | **No. of patients^†^** | **Reported MA model** | **Reported ES (95%CI)** | **Reported I^2^** | **Reported largest ES (95%CI) ^‡^** | **RA ES (95%CI)** | **RA I^2^** | **Egger’s p-value^¶^** | **AMSTAR 2** |
| --- | --- | --- | --- | --- | --- | --- | --- | --- | --- | --- | --- | --- | --- |
| Chen 2022 | Hilar cholangiocarcinoma | Biliary stenting with PDT | 1-year survival rate | 4 | 193 | Random | 0.56 (0.41-0.71) | NA | 0.39 (0.28-0.51) | 0.555 (0.406, 0.699) | 74 | 0.088 | CL |
| Chen 2022 | Hilar cholangiocarcinoma | Biliary stenting | 1-year survival rate | 4 | 176 | Random | 0.25 (0.16-0.33) | NA | 0.27 (0.16-0.37) | 0.258 (0.196, 0.325) | 34 | 0.670 | CL |
| Chen 2022 | Hilar cholangiocarcinoma | Biliary stenting with PDT | 2-year survival rate | 4 | 194 | Random | 0.16 (0.11-0.21) | NA | 0.14 (0.06-0.22) | 0.164 (0.116, 0.219) | 0 | 0.067 | CL |
| Chen 2022 | Hilar cholangiocarcinoma | Biliary stenting | 2-year survival rate | 4 | 179 | Random | 0.07 (0.03-0.11) | NA | 0.09 (0.03-0.16) | 0.076 (0.042, 0.119) | 0 | 0.727 | CL |
| Chen 2022 | Hilar cholangiocarcinoma | Biliary stenting with PDT | 3-year survival rate | 1 | 72 | Random | 0.04 (-0.00-0.09) | NA | 0.04 (-0.00-0.09) | 0.042 (0.009, 0.117) | NA | NA | CL |
| Chen 2022 | Hilar cholangiocarcinoma | Biliary stenting | 3-year survival rate | 1 | 71 | Random | 0.00 (-0.01-0.02) | NA | 0.00 (-0.01-0.02) | 0 (0.000, 0.051) | NA | NA | CL |
| Wang 2019 | Prostate cancer | PDT | Biopsy-negative rate | 9 | 574 | Random | 0.55 (0.44-0.66) | 85.7 (<0.001) | 0.76 (0.66-0.85) | 0.550 (0.417, 0.680) | 86 | 0.074 | CL |
| Wang 2019 | Prostate cancer | PDT | PSA decreasing rate (single arm) | 10 | 496 | Random | 0.32 (0.19-0.45) | 87 (<0.0001) | 0.38 (0.29-0.46) | 0.325 (0.224, 0.436) | 72 | 0.379 | CL |
| Wang 2019 | Prostate cancer | PDT | PSA decreasing rate (two arm) | 2 | 137 | Random | 0.49 (0.42-0.55) | 88.7 (<0.0001) | 0.48 (0.42-0.55) | 0.489 (0.406, 0.573) | 0 | NA | CL |
| Wang 2019 | Prostate cancer | PDT | PSA decreasing rate total | 12 | 633 | Random | 0.35 (0.24-0.47) | 71 | 0.38 (0.29-0.46) | 0.357 (0.264, 0.455) | 72 | 0.369 | CL |
| Guo 2021 | Prostate cancer | Vascular target PDT | Positive biopsy | 7 | 733 | Random | 0.36 (0.29-0.44) | 77 (<0.0001) | 0.5 (0.43-0.56) | 0.365 (0.294, 0.439) | 75 | 0.161 | L |
| Guo 2021 | Prostate cancer | PDT | Failure-free survival | 3 | 402 | Random | 0.77 (0.68-0.85) | 80.6 (0.006) | 0.84 (0.79-0.89) | 0.771 (0.684, 0.847) | 75 | 0.270 | L |
| Lansbury 2013 | SCC | PDT | Complete response | 14 | 273 | Fixed | 0.72 (0.669-0.768) | 71 | 0.744 (0.59-0.87) | 0.717 (0.541, 0.865) | 80 | 0.989 | H |
| Lansbury 2013 | SCC | PDT | Recurrence | 8 | 119 | Random | 0.26 (0.12-0.44) | 72 | 0.43 (0.18-0.44) | 0.165 (0.025, 0.394) | 88 | 0.009 | H |
| Spratt 2014 | Cutaneous Metastases from Advanced Cancer | PDT | Complete response | 5 | 40 | Random | 0.678 (0.389-0.875) | 90.40(<0.001) | 0.919 (0.84-0.96) | 0.710 (0.326, 0.970) | 94 | 0.415 | L |
| Spratt 2014 | Cutaneous Metastases from Advanced Cancer | PDT | Objective response | 5 | 40 | Random | 0.837 (0.562-0.954) | 82.02 (<0.001) | 0.994 (0.92-1.00) | 0.868 (0.560, 1.000) | 94 | 0.179 | L |
| Spratt 2014 | Cutaneous Metastases from Advanced Cancer | PDT | Recurrence rate | 2 | 13 | Fixed | 0.022 (0.001-0.248) | NA | 0.006 (0.00-0.085) | 0 (0.000, 0.011) | 0 | NA | L |
| Lin 2021 | Oral Squamous Cell Carcinoma | PDT | Complete response | 18 | 900 | Random | 0.799 (0.708-0.867) | 80.03 (<0.001) | 0.997 (0.96-1.00) | 0.810 (0.711, 0.893) | 92 | 0.188 | L |
| Lin 2021 | Oral Squamous Cell Carcinoma | PDT | Overall response | 7 | 507 | Random | 0.967 (0.902-0.959) | 55.69 (0.035) | 0.997 (0.96-1.00) | 0.988 (0.949, 1.000) | 85 | 0.744 | L |
| Lin 2021 | Oral Squamous Cell Carcinoma | PDT | Recurrence rate | 9 | 376 | Random | 0.158 (0.090-0.264) | 67.32 (0.002) | 0.068 (0.04-0.11) | 0.230 (0.061, 0.465) | 90 | 0.100 | L |
| Li 2023 | Non-muscle-invasive bladder cancer | Therapeutic PDT (intravenous) | Complete response | 13 | 300 | Random | 0.70 (0.60-0.81) | 84 (<0.01) | 0.74 (0.56-0.87) | 0.719 (0.592, 0.831) | 78 | 0.043 | CL |
| Li 2023 | Non-muscle-invasive bladder cancer | Therapeutic PDT (intravesical) | Complete response | 4 | 39 | Random | 0.60 (0.43-0.76) | 12 (0.33) | 0.75 (0.43-0.95) | 0.591 (0.423, 0.748) | 5 | 0.484 | CL |
| Li 2023 | Non-muscle-invasive bladder cancer with Tis | Therapeutic PDT (intravenous) | Complete response | 6 | 104 | Random | 0.69 (0.55-0.84) | 66 (0.01) | 0.69 (0.49-0.85) | 0.684 (0.526, 0.823) | 59 | 0.266 | CL |
| Li 2023 | Non-muscle-invasive bladder cancer with Tis | Therapeutic PDT (intravesical) | Complete response | 3 | 18 | Random | 0.64 (0.42-0.85) | 0 (0.50) | 0.68 (0.56-0.80) | 0.616 (0.387, 0.821) | 0 | 0.851 | CL |
| Li 2023 | Non-muscle-invasive bladder cancer | Adjuvant PDT | 6 months recurrence-free rate | 9 | 234 | Random | 0.95 (0.89-0.99) | 42 (0.08) | 0.95 (0.89-0.99) | 0.930 (0.869, 0.973) | 48 | 0.258 | CL |
| Li 2023 | Non-muscle-invasive bladder cancer | Adjuvant PDT | 12 months recurrence-free rate | 8 | 165 | Random | 0.79 (0.70-0.88) | 46 (0.07) | 0.78 (0.63-0.89) | 0.777 (0.676, 0.864) | 45 | 0.509 | CL |
| Li 2023 | Non-muscle-invasive bladder cancer | Adjuvant PDT | 24 months recurrence-free rat | 9 | 248 | Random | 0.56 (0.41-0.71) | 85 (<0.01) | 0.76 (0.65-0.84) | 0.567 (0.411, 0.716) | 80 | 0.139 | CL |
| Li 2023 | Non-muscle-invasive bladder cancer | Therapeutic PDT | 12 months recurrence-free rate | 10 | 107 | Random | 0.71 (0.56-0.85) | 70 (<0.01) | 0.48 (0.27-0.69) | 0.725 (0.547, 0.872) | 68 | 0.169 | CL |
| Li 2023 | Non-muscle-invasive bladder cancer | Therapeutic PDT | 24 months recurrence-free rate | 6 | 63 | Random | 0.38 (0.12-0.64) | 89 (0.0879) | 0.22 (0.06-0.48) | 0.358 (0.105, 0.665) | 83 | 0.652 | CL |
| Li 2023 | Non-muscle-invasive bladder cancer with Tis | Therapeutic PDT | 12 months recurrence-free rate | 5 | 25 | Random | 0.84 (0.48-1.00) | 61 (0.04) | 0.42 (0.15-0.73) | 0.888 (0.527, 1.000) | 78 | 0.238 | CL |
| Li 2023 | Non-muscle-invasive bladder cancer with Tis | Therapeutic PDT | 24 months recurrence-free rate | 5 | 29 | Random | 0.15 (0.00-0.42) | 36 (0.18) | 0.23 (0.05-0.54) | 0.153 (0.005, 0.448) | 62 | 0.873 | CL |
| Li 2023 | BCG-unresponsive non-muscle-invasive bladder cancer | Adjuvant PDT | 6 months recurrence-free rate | 3 | 42 | Random | 0.93 (0.78-1.00) | 4 (0.35) | 0.97 (0.85-1.00) | 0.898 (0.681, 0.997) | 40 | 0.011 | CL |
| Li 2023 | BCG-unresponsive non-muscle-invasive bladder cancer | Adjuvant PDT | 12 months recurrence-free rate | 6 | 95 | Random | 0.68 (0.51-0.86) | 74 (<0.01) | 0.91 (0.76-0.98) | 0.685 (0.492, 0.850) | 71 | 0.343 | CL |
| Li 2023 | BCG-unresponsive non-muscle-invasive bladder cancer | Adjuvant PDT | 24 months recurrence-free rate | 4 | 82 | Random | 0.56 (0.32-0.81) | 83 (<0.01) | 0.71 (0.53-0.85) | 0.567 (0.315, 0.802) | 80 | 0.770 | CL |

**^ƒ^** In the original meta-analysis, the number of included studies on specific intervention measures and outcomes.

**^†^** In the original meta-analysis, the number of patients (intervention/comparison) on specific intervention measures and outcomes.

**^‡^** In the original meta-analysis, the effect size of the clinical study with the largest sample size.

**^£^**The original meta-analysis should assess whether the statistical significance of the effect size was consistent across individual clinical studies.

**^¶^** The p-value of the egger test for the meta-analysis, which reflects publication bias or selective reporting bias.

Abbreviation: AMSTAR 2, assessment of multiple systematic reviews; BCG, Bacille Calmette-Guérin; CL, critical low; CI, confidence interval; ES, effect size; H, high; L, low; MA, meat-analysis; NA, not available; PDT, photodynamic therapy; RA, re-analyze; SCC, squamous cell carcinoma.
