# Supplementary material for: Umbrella review of photodynamic therapy for cancer: efficacy, safety, and clinical applications
Source: Front Oncol. 2025 Aug 4;15:1528314. doi: 10.3389/fonc.2025.1528314 (PMC12358287; doi:10.3389/fonc.2025.1528314)
Supplement: Supplementary Table 7 — Detailed results of subgroup meta-analyses association. [file Table7.docx]

Table S7. Detailed results of subgroup meta-analysis association.

| **Author** | **Cancer** | **Intervention** | **Comparison** | **Outcome** | **No. studies ^ƒ^** | **No. of patients^†^** | **Metric** | **Reported MA model** | **Reported p-value** | **Reported ES (95%CI)** | **Reported I^2^** | **ES (95%CI) of largest study^‡^** | **Consistence of individual study (Y/N) ^£^** | **RA model** | **RA ES (95%CI)** | **P value^§^** | **PI ES (95%)** | **RA I^2^** | **Egger p-value^¶^** | **TES p-value^€^** | **Class** | **AMSTAR 2** |
| --- | --- | --- | --- | --- | --- | --- | --- | --- | --- | --- | --- | --- | --- | --- | --- | --- | --- | --- | --- | --- | --- | --- |
| 1. Subgroup analysis of OS of unresectable cholangiocarcinoma treated by PDT according to study type. | | | | | | | | | | | | | | | | | | | | | | |
| Lu 2015 | Unresectable cholangiocarcinoma | Biliary stenting with PDT | Biliary stenting | OS (RCT) | 2 | 36/35 | HR | Random | <0.0001 | 0.25 (0.15, 0.43) | 18 (0.27) | 0.21 (0.12, 0.37) | Y | HKSJ | 0.255 (0.008, 7.716) | 0.123 | NA | 16.353 | NA | NA | ns | CL |
| Lu 2015 | Unresectable cholangiocarcinoma | Biliary stenting with PDT | Biliary stenting | OS (non-RCT) | 5 | 230/301 | HR | Random | 0.001 | 0.67 (0.52, 0.85) | 13 (0.33) | 0.79 (0.51, 1.22) | N | DL | 0.666 (0.523, 0.849) | 0.001 | (0.399, 1.112) | 13.239 | 0.286 | 0.399 | IV | CL |
| 2. Subgroup analysis of 1-year recurrence rate of basal cell carcinoma treated by PDT according to intervention. | | | | | | | | | | | | | | | | | | | | | | |
| Ou-yang 2023 | Basal cell carcinoma | MAL-PDT | ALA-PDT | 1-year recurrence | 2 | 168/182 | RR | Random | 0.39 | 1.60 (0.55, 4.66) | 48 (0.17) | 1.01 (0.41, 2.52) | Y | HKSJ | 1.314 (0.078, 22.025) | 0.435 | NA | 46.811 | NA | NA | ns | L |
| Wang 2015 | Basal cell carcinoma | PDT | Cryotherapy | 1-year recurrence | 2 | 144/132 | RR | Random | 0.92 | 1.04 (0.46, 2.39) | 47 (0.17) | 0.70 (0.31, 1.58) | Y | HKSJ | 1.048 (0.054, 20.259) | 0.875 | NA | 47.193 | NA | NA | ns | CL |
| Wang 2020 | Basal cell carcinoma | MAL-PDT | Imiquimod | 1-year recurrence | 1 | 196/189 | RR | Random | NA | 1.62 (1.09, 2.41) | NA | 1.62 (1.09, 2.41) | NA | HKSJ | 1.314 (1.072, 1.611) | 0.009 | NA | NA | NA | NA | IV | CL |
| Wang 2020 | Basal cell carcinoma | MAL-PDT | Fluorouracil | 1-year recurrence | 1 | 196/198 | RR | Random | NA | 1.35 (0.93, 1.94) | NA | 1.35 (0.93, 1.94) | NA | HKSJ | 1.202 (0.971, 1.489) | 0.091 | NA | NA | NA | NA | ns | CL |
| Wang 2015 | Basal cell carcinoma | MAL-PDT | Pharmacologic treatment | 1-year recurrence | 1 | 156/334 | RR | Random | 0.06 | 1.67 (0.97, 2.85) | NA | 1.67 (0.97, 2.85) | NA | HKSJ | 1.432 (1.009, 2.033) | 0.044 | NA | NA | NA | NA | IV | CL |
| Ou-yang 2023 | Basal cell carcinoma | MAL-PDT | Surgery | 1-year recurrence | 1 | 53/52 | RR | Random | 0.07 | 6.87 (0.88, 53.89) | NA | 6.87 (0.88, 53.89) | NA | HKSJ | 1.845 (1.319, 2.58) | <0.001 | NA | NA | NA | NA | IV | L |
| Ou-yang 2023 | Basal cell carcinoma | ALA-PDT | Surgery | 1-year recurrence | 1 | 21/19 | RR | Random | 0.16 | 10.00 (0.59, 169.63) | NA | 10.00 (0.59, 169.63) | NA | HKSJ | 2 (1.302, 3.072) | 0.002 | NA | NA | NA | NA | IV | L |
| 3. Subgroup analysis of 2-year recurrence rate of basal cell carcinoma treated by PDT according to intervention. | | | | | | | | | | | | | | | | | | | | | | |
| Ou-yang 2023 | Basal cell carcinoma | MAL-PDT | Surgery | 2-year recurrence | 1 | 53/53 | RR | Random | 0.03 | 5.00 (1.15, 21.74) | NA | 5.00 (1.15, 21.74) | NA | HKSJ | 1.822 (1.303, 2.548) | <0.001 | NA | NA | NA | NA | IV | L |
| 4. Subgroup analysis of 3-year recurrence rate of basal cell carcinoma treated by PDT according to intervention. | | | | | | | | | | | | | | | | | | | | | | |
| Ou-yang 2023 | Basal cell carcinoma | ALA-PDT | Surgery | 3-year recurrence | 1 | 83/88 | RR | Random | 0.0009 | 11.13 (2.69, 46.01) | NA | 11.13 (2.69, 46.01) | NA | HKSJ | 2.18 (1.735, 2.737) | <0.001 | NA | NA | NA | NA | IV | L |
| Ou-yang 2023 | Basal cell carcinoma | MAL-PDT | Fluorouracil | 3-year recurrence | 1 | 126/146 | RR | Random | 0.17 | 1.36 (0.88, 2.10) | NA | 1.36 (0.88, 2.10) | NA | HKSJ | 1.226 (0.932, 1.613) | 0.146 | NA | NA | NA | NA | ns | L |
| Ou-yang 2023 | Basal cell carcinoma | MAL-PDT | Imiquimod | 3-year recurrence | 1 | 126/145 | RR | Random | <0.0001 | 3.91 (2.02, 7.60) | NA | 3.91 (2.02, 7.60) | NA | HKSJ | 1.907 (1.523, 2.387) | <0.001 | NA | NA | NA | NA | IV | L |
| 5. Subgroup analysis of 5-year recurrence rate of basal cell carcinoma treated by PDT according to intervention. | | | | | | | | | | | | | | | | | | | | | | |
| Zou 2016 | Basal cell carcinoma | PDT | Surgery | 5-year recurrence | 2 | 136/140 | RR | Fixed | <0.0001 | 8.25 (3.01, 22.62) | 41 (0.19) | 13.25 (3.24, 22.62) | N | HKSJ | 2.016 (0.237, 17.188) | 0.150 | NA | 53.492 | NA | NA | ns | L |
| Ou-yang 2023 | Basal cell carcinoma | MAL-PDT | Surgery | 5-year recurrence | 1 | 49/52 | RR | Random | 0.09 | 3.71 (0.81, 17.02) | NA | 3.71 (0.81, 17.02) | NA | HKSJ | 1.704 (1.126, 2.578) | 0.012 | NA | NA | NA | NA | IV | L |
| Wang 2020 | Basal cell carcinoma | MAL-PDT | Cryotherapy | 5-year recurrence | 1 | 100/93 | RR | Random | NA | 1.08 (0.62, 1.86) | NA | 1.08 (0.62, 1.86) | NA | HKSJ | 1.046 (0.756, 1.446) | 0.787 | NA | NA | NA | NA | ns | CL |
| Wang 2020 | Basal cell carcinoma | MAL-PDT | Imiquimod | 5-year recurrence | 1 | 196/189 | RR | Random | NA | 1.88 (1.32, 2.66) | NA | 1.88 (1.32, 2.66) | NA | HKSJ | 1.462 (1.212, 1.765) | <0.001 | NA | NA | NA | NA | IV | CL |
| Wang 2020 | Basal cell carcinoma | MAL-PDT | Fluorouracil | 5-year recurrence | 1 | 196/198 | RR | Random | NA | 1.24 (0.93, 1.66) | NA | 1.24 (0.93, 1.66) | NA | HKSJ | 1.168 (0.955, 1.429) | 0.132 | NA | NA | NA | NA | ns | CL |
| 6. Subgroup analysis of cosmetic outcome of basal cell carcinoma treated by PDT according to intervention. | | | | | | | | | | | | | | | | | | | | | | |
| Wang 2020 | Basal cell carcinoma | MAL-PDT | Surgery | Cosmetic outcome | 2 | 127/131 | RR | Random | <0.0001 | 1.99 (1.50, 2.63) | 36 (0.21) | 1.81 (1.46, 2.25) | Y | HKSJ | 3.988 (2.443, 6.509) | <0.001 | NA | 0 | NA | NA | IV | CL |
| Ou-yang 2023 | Basal cell carcinoma | MAL-PDT | Surgery | 3 months cosmetic outcome | 2 | 172/161 | RR | Random | 0.01 | 1.12 (1.03, 1.22) | NA | 1.12 (1.03, 1.22) | Y | HKSJ | 1.589 (1.040, 2.428) | 0.032 | NA | 0 | NA | NA | IV | L |
| Ou-yang 2023 | Basal cell carcinoma | MAL-PDT | Surgery | 12 months cosmetic outcome | 2 | 170/160 | RR | Random | 0.0002 | 1.24 (1.10, 1.38) | 29 (0.23) | 1.30 (1.15, 1.47) | Y | HKSJ | 2.693 (1.580, 4.591) | <0.001 | NA | 0 | NA | NA | IV | L |
| Wang 2015 | Basal cell carcinoma | MAL-PDT | Cryotherapy | Cosmetic outcome | 2 | 142/130 | RR | Random | <0.0001 | 1.51 (1.30, 1.76) | 0 (0.35) | 1.45 (1.22, 1.73) | Y | HKSJ | 2.897 (1.799, 4.664) | <0.001 | NA | 0 | NA | NA | IV | CL |
| Wang 2015 | Basal cell carcinoma | MAL-PDT | Pharmacologic treatment | Cosmetic outcome | 1 | 186/377 | RR | Random | 0.5 | 1.05 (0.91, 1.21) | NA | 1.05 (0.91, 1.21) | NA | HKSJ | 1.087 (0.852, 1.387) | 0.503 | NA | NA | NA | NA | ns | CL |
| Wang 2020 | Basal cell carcinoma | MAL-PDT | ALA-PDT | Cosmetic outcome | 1 | 72/73 | RR | Random | NA | 0.84 (0.69, 1.03) | NA | 0.84 (0.69, 1.03) | NA | HKSJ | 0.736 (0.532, 1.018) | 0.064 | NA | NA | NA | NA | ns | CL |
| Wang 2020 | Basal cell carcinoma | MAL-PDT | PDT with BF-200 ALA | Cosmetic outcome | 1 | 57/56 | RR | Random | NA | 0.94 (0.81, 1.10) | NA | 0.94 (0.81, 1.10) | NA | HKSJ | 0.832 (0.532, 1.302) | 0.421 | NA | NA | NA | NA | ns | CL |
| Wang 2020 | Basal cell carcinoma | MAL-PDT | Placebo | Cosmetic outcome | 1 | 43/15 | RR | Random | NA | 1.05 (0.91, 1.21) | NA | 1.05 (0.91, 1.21) | NA | HKSJ | 1.500 (0.372, 6.047) | 0.569 | NA | NA | NA | NA | ns | CL |
| Wang 2020 | Basal cell carcinoma | MAL-PDT | Imiquimod | Cosmetic outcome | 1 | 186/184 | RR | Random | NA | 1.02 (0.87, 1.19) | NA | 1.02 (0.87, 1.19) | NA | HKSJ | 1.020 (0.827, 1.258) | 0.851 | NA | NA | NA | NA | ns | CL |
| Wang 2020 | Basal cell carcinoma | MAL-PDT | Fluorouracil | Cosmetic outcome | 1 | 186/193 | RR | Random | NA | 1.08 (0.92, 1.28) | NA | 1.08 (0.92, 1.28) | NA | HKSJ | 1.110 (0.896, 1.374) | 0.341 | NA | NA | NA | NA | ns | CL |
| Ou-yang 2023 | Basal cell carcinoma | MAL-PDT | HAL-PDT | 3 months cosmetic outcome | 1 | 31/31 | RR | Random | 0.18 | 1.26 (0.90, 1.77) | NA | 1.26 (0.90, 1.77) | NA | HKSJ | 1.515 (0.794, 2.89) | 0.208 | NA | NA | NA | NA | ns | L |
| 7. Subgroup analysis of complete clearance of basal cell carcinoma treated by PDT according to intervention. | | | | | | | | | | | | | | | | | | | | | | |
| Wang 2015 | Basal cell carcinoma | PDT | Surgery | Complete clearance | 4 | 282/273 | RR | Random | 0.003 | 0.93 (0.89, 0.98) | 19 (0.30) | 0.93 (0.88, 0.98) | N | HKSJ | 0.563 (0.493, 0.643) | 0.001 | (0.471, 0.674) | 0 | 0.626 | 0.543 | IV | CL |
| Wang 2015 | Basal cell carcinoma | MAL-PDT | Cryotherapy | Complete clearance | 1 | 114/98 | RR | Random | 0.06 | 0.92 (0.85, 1.00) | NA | 0.92 (0.85, 1.00) | NA | HKSJ | 0.703 (0.52, 0.95) | 0.022 | NA | NA | NA | NA | IV | CL |
| Wang 2015 | Basal cell carcinoma | MAL-PDT | Topical therapy (imiquimod and fluorouracil) | Complete clearance | 1 | 196/387 | RR | Random | 0.13 | 0.95 (0.88, 1.02) | NA | 0.95 (0.88, 1.02) | NA | HKSJ | 0.774 (0.575, 1.041) | 0.090 | NA | NA | NA | NA | ns | CL |
| Wang 2015 | Basal cell carcinoma | MAL-PDT | Placebo | Complete clearance | 1 | 75/75 | RR | Random | <0.0001 | 2.75 (1.84, 4.10) | NA | 2.75 (1.84, 4.10) | NA | HKSJ | 2.750 (1.845, 4.1) | <0.001 | NA | NA | NA | NA | IV | CL |
| 8. Subgroup analysis of 3 months complete response of basal cell carcinoma treated by PDT according to intervention. | | | | | | | | | | | | | | | | | | | | | | |
| Wang 2020 | Basal cell carcinoma | MAL-PDT | Surgery | 3 months complete response | 2 | 181/170 | RR | Random | 0.002 | 0.93 (0.89, 0.97) | 0 (0.90) | 0.93 (0.88, 0.98) | N | HKSJ | 0.559 (0.423, 0.739) | 0.024 | NA | 0 | NA | NA | IV | CL |
| Ou-yang 2023 | Basal cell carcinoma | MAL-PDT | ALA-PDT | 3 months complete response | 3 | 220/233 | RR | Random | 0.67 | 0.99 (0.94, 1.04) | 0 (0.85) | 0.99 (0.94, 1.04) | Y | HKSJ | 0.916 (0.67, 1.251) | 0.349 | (0.364, 2.303) | 0 | 0.357 | 0.647 | ns | L |
| Wang 2020 | Basal cell carcinoma | MAL-PDT | Placebo | 3 months complete response | 1 | 75/75 | RR | Random | <0.0001 | 2.75 (1.84, 4.10) | NA | 2.75 (1.84, 4.10) | NA | HKSJ | 2.750 (1.845, 4.100) | <0.001 | NA | NA | NA | NA | IV | CL |
| Wang 2020 | Basal cell carcinoma | MAL-PDT | Cryotherapy | 3 months complete response | 1 | 103/98 | RR | Random | 0.43 | 1.02 (0.97, 1.08) | NA | 1.02 (0.97, 1.08) | NA | HKSJ | 1.382 (0.559, 3.415) | 0.484 | NA | NA | NA | NA | ns | CL |
| Wang 2020 | Basal cell carcinoma | MAL-PDT | Imiquimod | 3 months complete response | 1 | 196/189 | RR | Random | 0.09 | 0.94 (0.87, 1.01) | NA | 0.94 (0.87, 1.01) | NA | HKSJ | 0.794 (0.623, 1.013) | 0.063 | NA | NA | NA | NA | ns | CL |
| Wang 2020 | Basal cell carcinoma | MAL-PDT | Fluorouracil | 3 months complete response | 1 | 196/198 | RR | Random | 0.29 | 0.96 (0.88, 1.04) | NA | 0.96 (0.88, 1.04) | NA | HKSJ | 0.864 (0.668, 1.117) | 0.263 | NA | NA | NA | NA | ns | CL |
| Ou-yang 2023 | Basal cell carcinoma | MAL-PDT | HAL-PDT | 3 months complete response | 1 | 31/31 | RR | Random | 0.39 | 1.07 (0.91, 1.27) | NA | 1.07 (0.91, 1.27) | NA | HKSJ | 1.554 (0.487, 4.953) | 0.456 | NA | NA | NA | NA | ns | L |
| 9. Subgroup analysis of 1-year complete response of basal cell carcinoma treated by PDT according to intervention. | | | | | | | | | | | | | | | | | | | | | | |
| Zou 2016 | Basal cell carcinoma | PDT | Surgery | 1-year complete response | 3 | 157/159 | RR | Fixed | 0.006 | 0.89 (0.80, 0.99) | 0 (0.47) | 0.88 (0.76, 1.02) | Y | HKSJ | 0.646 (0.544, 0.767) | 0.008 | (0.389, 1.073) | 0 | 0.047 | 0.710 | IV | L |
| Wang 2020 | Basal cell carcinoma | MAL-PDT | Surgery | 1-year complete response | 2 | 171/169 | RR | Random | 0.0002 | 0.90 (0.85, 0.95) | 0 (0.48) | 0.91 (0.85, 0.96) | Y | HKSJ | 0.513 (0.252, 1.043) | 0.053 | NA | 0 | NA | NA | ns | CL |
| Ou-yang 2023 | Basal cell carcinoma | MAL-PDT | ALA-PDT | 1-year complete response | 2 | 168/182 | RR | Random | 0.34 | 0.96 (0.88, 1.05) | 45 (0.18) | 1.00 (0.92, 1.09) | Y | HKSJ | 0.761 (0.045, 12.763) | 0.435 | NA | 46.811 | NA | NA | ns | L |
| Wang 2020 | Basal cell carcinoma | MAL-PDT | Cryotherapy | 1-year complete response | 1 | 100/93 | RR | Random | NA | 1.04 (0.95, 1.15) | NA | 1.04 (0.95, 1.15) | NA | HKSJ | 1.234 (0.739, 2.063) | 0.421 | NA | NA | NA | NA | ns | CL |
| Wang 2020 | Basal cell carcinoma | MAL-PDT | Imiquimod | 1-year complete response | 1 | 156/165 | RR | Random | NA | 0.93 (0.87, 1.01) | NA | 0.93 (0.87, 1.01) | NA | HKSJ | 0.737 (0.554, 0.980) | 0.036 | NA | NA | NA | NA | IV | CL |
| Wang 2020 | Basal cell carcinoma | MAL-PDT | Fluorouracil | 1-year complete response | 1 | 156/169 | RR | Random | NA | 0.95 (0.88, 1.03) | NA | 0.95 (0.88, 1.03) | NA | HKSJ | 0.801 (0.592, 1.083) | 0.150 | NA | NA | NA | NA | ns | CL |
| 10. Subgroup analysis of AEs of basal cell carcinoma treated by PDT according to intervention. | | | | | | | | | | | | | | | | | | | | | | |
| Wang 2020 | Basal cell carcinoma | MAL-PDT | Surgery | AEs | 2 | 152/145 | RR | Random | <0.0001 | 2.12 (1.46, 3.09) | 0 (0.38) | 2.54 (1.47, 4.39) | Y | HKSJ | 1.641 (1.187, 2.269) | 0.033 | NA | 0 | NA | NA | IV | CL |
| Wang 2020 | Basal cell carcinoma | MAL-PDT | ALA-PDT | AEs | 1 | 143/138 | RR | Random | NA | 1.00 (0.99, 1.01) | NA | 1.00 (0.99, 1.01) | NA | HKSJ | 1.018 (0.143, 7.249) | 0.986 | NA | NA | NA | NA | ns | CL |
| Wang 2020 | Basal cell carcinoma | MAL-PDT | Placebo | AEs | 1 | 66/65 | RR | Random | NA | 1.37 (1.14, 1.66) | NA | 1.37 (1.14, 1.66) | NA | HKSJ | 2.718 (1.313, 5.629) | 0.007 | NA | NA | NA | NA | IV | CL |
| Wang 2020 | Basal cell carcinoma | MAL-PDT | Cryotherapy | AEs | 1 | 60/58 | RR | Random | NA | 0.92 (0.76, 1.13) | NA | 0.92 (0.76, 1.13) | NA | HKSJ | 0.856 (0.583, 1.256) | 0.426 | NA | NA | NA | NA | ns | CL |
| 11. Subgroup analysis of lesion reduction of Bowen’s disease treated by PDT according to intervention. | | | | | | | | | | | | | | | | | | | | | | |
| Zhong 2020 | Bowen’s disease | PDT | 5-Fu | Lesion reduction | 4 | 176/88 | OR | Fixed | <0.0001 | 3.70 (2.07, 6.62) | 60 (0.06) | 2.00 (0.84, 4.78) | N | HKSJ | 5.266 (0.578, 47.994) | 0.097 | (0.045, 622.621) | 59.131 | 0.021 | 0.003 | ns | L |
| Zhong 2020 | Bowen’s disease | PDT | Cryotherapy | Lesion reduction | 2 | 144/111 | OR | Fixed | 0.008 | 2.24 (1.24, 4.40) | 0 (0.63) | 2.08 (1.07, 4.03) | Y | HKSJ | 2.234 (0.353, 14.152) | 0.114 | NA | 0 | NA | NA | ns | L |
| 12. Subgroup analysis of recurrence rate of Bowen’s disease treated by PDT according to intervention. | | | | | | | | | | | | | | | | | | | | | | |
| Zhong 2020 | Bowen’s disease | PDT | 5-Fu | Recurrence rate | 2 | 157/69 | OR | Fixed | 0.41 | 0.69 (0.28, 1.69) | 39 (0.20) | 1.10 (0.34, 3.55) | Y | HKSJ | 0.652 (0, 2553.233) | 0.630 | NA | 38.476 | NA | NA | ns | L |
| Zhong 2020 | Bowen’s disease | PDT | Cryotherapy | Recurrence rate | 1 | 124/73 | OR | Fixed | 0.11 | 0.53 (0.24, 1.16) | NA | 0.53 (0.24, 1.16) | NA | HKSJ | 0.532 (0.243, 1.165) | 0.114 | NA | NA | NA | NA | ns | L |
| 13. Subgroup analysis of complete response rate of Bowen’s disease treated by PDT according to the line of therapy. | | | | | | | | | | | | | | | | | | | | | | |
| Xue 2021 | Bowen's Disease | PDT | 5-FU, cryotherapy | Complete response rate (after first treatment) | 2 | 53/53 | RR | Random | 0.006 | 1.66 (1.16, 2.37) | 0 (0.58) | 1.83 (1.10, 3.06) | N | HKSJ | 1.856 (1.423, 2.42) | 0.022 | NA | 0 | NA | NA | IV | CL |
| Xue 2021 | Bowen's Disease | PDT | 5-FU, cryotherapy | Complete response rate (after second treatment) | 2 | 53/53 | RR | Random | 0.38 | 1.28 (0.84, 1.97) | 0 (0.79) | 0.70 (0.30, 1.62) | Y | HKSJ | 0.818 (0.375, 1.783) | 0.188 | NA | 0 | NA | NA | ns | CL |

**^ƒ^** In the original meta-analysis, the number of included studies on specific intervention measures and outcomes.

**^†^** In the original meta-analysis, the number of patients (intervention/comparison) on specific intervention measures and outcomes.

**^‡^** In the original meta-analysis, the effect size of the clinical study with the largest sample size.

**^£^**The original meta-analysis should assess whether the statistical significance of the effect size was consistent across individual clinical studies.

**^§^** The p-value resynthesized from the original meta-analysis.

**^¶^** The p-value of the egger test for the meta-analysis, which reflects publication bias or selective reporting bias.

**^€^** This is the P-value of the excess significance test for the meta-analysis, which reflects publication bias or selective reporting bias.

Abbreviation: AEs, Adverse events; ALA, 5-Aminolevulinic acid; AMSTAR 2, assessment of multiple systematic reviews; BCC, basal cell carcinoma; CL, critical low; CI, confidence interval; DL, DerSimonian-Laird; ES, effect size; HKSJ, Hartung-Knapp-Sidik-Jonkman; HR, hazard ratio; L, low; MA, meat-analysis; MAL, methyl aminolevulinate; N, no; NA, not available; OR, odds ratio; OS, overall survival; PDT, photodynamic therapy; PI, prediction interval; RA, re-analyze; RR, risk ratio; SCC, squamous cell carcinoma; TES, test of excess significance; WMD, weighted mean difference; Y, yes; YAG-AFL, erbium: yttrium-aluminum-garnet ablative factional laser; 5-FU, 5-Fluorouracil; IV, weak evidence (class IV); ns, non-significant (class ns).
