# Supplementary material for: Umbrella review of photodynamic therapy for cancer: efficacy, safety, and clinical applications
Source: Front Oncol. 2025 Aug 4;15:1528314. doi: 10.3389/fonc.2025.1528314 (PMC12358287; doi:10.3389/fonc.2025.1528314)
Supplement: Supplementary Table 8 — Citation matrices for meta-analyses with overlapping associations. [file Table8.docx]

Table S8. Citation matrices for meta-analyses with overlapping associations.

**1. Overall survival of cholangiocarcinoma (Biliary stenting with PDT vs stenting alone)**

| **Meta-analysis** | Lu 2015 | Chen 2022 |
| --- | --- | --- |
| **Individual study** |  | |
| Ortner 2003 | 1 | 0 |
| Zoepf 2005 | 1 | 0 |
| Witzgmann 2006 | 1 | 0 |
| Kahaleh 2008 | 1 | 0 |
| Matull 2011 | 1 | 0 |
| Weigt 2011 | 1 | 0 |
| Cheon 2012 | 1 | 1 |
| Li 2020 | 0 | 1 |
| **Decision to retain** | ✓ | ✓ |
| **Grand Total (N)** | 9 | |
| **Rows (r)** | 8 | |
| **Columns (c)** | 2 | |
| **CCA (N-r/rc-r)** | 12.5% | |

**2. 1-year recurrence risk of basal cell carcinoma (PDT vs surgery)**

| **Meta-analysis** | Wang 2015 | Zou 2016 | Wang 2020 |
| --- | --- | --- | --- |
| **Individual study** |  | | |
| Rhodes 2007 | 1 | 0 | 1 |
| Roozeboom 2013 | 1 | 0 | 0 |
| Szeimies 2008 | 1 | 0 | 1 |
| Laura Berroeta 2007 | 0 | 1 | 0 |
| Lesley E 2007 | 0 | 1 | 0 |
| Lesly E 2004 | 0 | 1 | 0 |
| Marieke H 2013 | 0 | 1 | 0 |
| **Decision to retain** | ✓ | ✓ | ✘ |
| **Grand Total (N)** | 9 | | |
| **Rows (r)** | 7 | | |
| **Columns (c)** | 3 | | |
| **CCA (N-r/rc-r)** | 14.2% | | |

**3. 1-year recurrence risk of basal cell carcinoma (PDT vs surgery)**

| **Meta-analysis** | Wang 2015 | Wang 2020 |
| --- | --- | --- |
| **Individual study** |  | |
| Rhodes 2007 | 1 | 1 |
| Roozeboom 2013 | 1 | 0 |
| Szeimies 2008 | 1 | 1 |
| Basset-Seguin 2008 | 1 | 0 |
| Wang 2001 | 1 | 0 |
| Arits 2013 | 1 | 0 |
| Morton 2018 2018 | 0 | 1 |
| Arits 2013/Roozeboom 2014/Jansen 2017 | 0 | 1 |
| Arits 2013/Jansen 2017 | 0 | 1 |
| **Decision to retain** | ✘ | ✓ |
| **Grand Total (N)** | 11 | |
| **Rows (r)** | 9 | |
| **Columns (c)** | 2 | |
| **CCA (N-r/rc-r)** | 22.2% | |

**4. 1-year recurrence risk of basal cell carcinoma (PDT vs cryotherapy)**

| **Meta-analysis** | Wang 2015 | Wang 2020 |
| --- | --- | --- |
| **Individual study** |  | |
| Basset-Seguin 2008 | 1 | 1 |
| Wang 2001 | 1 | 0 |
| **Decision to retain** | ✓ | ✘ |
| **Grand Total (N)** | 3 | |
| **Rows (r)** | 2 | |
| **Columns (c)** | 2 | |
| **CCA (N-r/rc-r)** | 50.0% | |

**5. 5-year recurrence risk of basal cell carcinoma (PDT vs surgery)**

| **Meta-analysis** | Wang 2015 | Zou 2016 | Wang 2020 |
| --- | --- | --- | --- |
| **Individual study** |  | | |
| Rhodes 2007 | 1 | 0 | 1 |
| Roozeboom 2013 | 1 | 0 | 0 |
| Lesley E 2007 | 0 | 1 | 0 |
| Marieke H 2013 | 0 | 1 | 0 |
| **Decision to retain** | ✓ | ✓ | ✘ |
| **Grand Total (N)** | 5 | | |
| **Rows (r)** | 4 | | |
| **Columns (c)** | 3 | | |
| **CCA (N-r/rc-r)** | 12.5% | | |

**6. 5-year recurrence risk of basal cell carcinoma (overall)**

| **Meta-analysis** | Wang 2015 | Wang 2020 |
| --- | --- | --- |
| **Individual study** |  | |
| Rhodes 2007 | 1 | 1 |
| Roozeboom 2013 | 1 | 0 |
| Basset-Seguin 2008 | 1 | 1 |
| Arits 2013/Roozeboom 2014/Jansen 2017 2017 | 0 | 1 |
| Arits 2013/Jansen 2017 | 0 | 1 |
| **Decision to retain** | **✘** | **✓** |
| **Grand Total (N)** | 7 | |
| **Rows (r)** | 5 | |
| **Columns (c)** | 2 | |
| **CCA (N-r/rc-r)** | 40.0% | |

**7. Cosmetic outcome of basal cell carcinoma (PDT vs surgery)**

| **Meta-analysis** | Wang 2015 | Wang 2020 |
| --- | --- | --- |
| **Individual study** |  | |
| Rhodes 2007 | 1 | 0 |
| Roozeboom 2013 | 1 | 1 |
| Szeimies 2008 | 0 | 1 |
| **Decision to retain** | **✘** | **✓** |
| **Grand Total (N)** | 4 | |
| **Rows (r)** | 3 | |
| **Columns (c)** | 2 | |
| **CCA (N-r/rc-r)** | 33.3% | |

**8. Cosmetic outcome of basal cell carcinoma (overall)**

| **Meta-analysis** | Wang 2015 | Collier 2018 | Wang 2020 |
| --- | --- | --- | --- |
| **Individual study** |  | | |
| Rhodes 2007 | 1 | 0 | 1 |
| Roozeboom 2013 | 1 | 0 | 0 |
| Basset-Seguin 2008 | 1 | 1 | 1 |
| Wang 2001 | 1 | 1 | 0 |
| Arits 2013 | 1 | 0 | 0 |
| Szeimies 2008 | 0 | 0 | 1 |
| Kessels 2018 | 0 | 0 | 1 |
| Morton 2018 | 0 | 0 | 1 |
| Foley 2009 | 0 | 0 | 1 |
| Arits 2013/Roozeboom 2014/Jansen 2017 | 0 | 0 | 1 |
| Arits 2013/ Jansen 2017 | 0 | 0 | 1 |
| **Decision to retain** | ✘ | ✘ | ✓ |
| **Grand Total (N)** | 15 | | |
| **Rows (r)** | 11 | | |
| **Columns (c)** | 3 | | |
| **CCA (N-r/rc-r)** | 18.2% | | |

**9. Cosmetic outcome of basal cell carcinoma (PDT vs cryotherapy)**

| **Meta-analysis** | Wang 2015 | Wang 2020 |
| --- | --- | --- |
| **Individual study** |  | |
| Basset-Seguin 2008 | 1 | 1 |
| Wang2001 | 1 | 0 |
| **Decision to retain** | ✓ | ✘ |
| **Grand Total (N)** | 3 | |
| **Rows (r)** | 2 | |
| **Columns (c)** | 2 | |
| **CCA (N-r/rc-r)** | 50.0% | |

**10. Complete response of basal cell carcinoma (PDT vs surgery)**

| **Meta-analysis** | Zou 2016 | Wang 2020 |
| --- | --- | --- |
| **Individual study** |  | |
| Rhodes 2007 | 1 | 1 |
| Szeimies 2008 | 0 | 1 |
| Marieke H 2013 | 1 | 0 |
| **Decision to retain** | ✘ | ✓ |
| **Grand Total (N)*** | 4 | |
| **Rows (r)*** | 3 | |
| **Columns (c)*** | 2 | |
| **CCA (N-r/rc-r)** | 33.3% | |

* N is the total number of times primary publications appeared in reviews (inclusive of double-counting), r is the number of unique primary publications, and c is the number of systematic reviews included in the umbrella review.
