# Supplementary material for: Umbrella review of photodynamic therapy for cancer: efficacy, safety, and clinical applications
Source: Front Oncol. 2025 Aug 4;15:1528314. doi: 10.3389/fonc.2025.1528314 (PMC12358287; doi:10.3389/fonc.2025.1528314)
Supplement: Supplementary Table 9 — Recalculated detailed results of meta-analyses using the by trim-and-fill method. [file Table9.docx]

Table S9. Recalculated detailed results of meta-analyses using the by trim-and-fill method.

| **Author** | **Cancer** | **Intervention** | **Comparison** | **Outcome** | **No. of studies ^ƒ^** | **No. of patients^£^** | **RA ES (95%CI) ^‡^** | **RA p- value** | **Egger p-value^¶^** | **TES p-value^€^** | **Class** | **AMSTAR 2** | **TMFA ES (95%CI)** | **TMFA p-value** | **No. of added studies^§^** |
| --- | --- | --- | --- | --- | --- | --- | --- | --- | --- | --- | --- | --- | --- | --- | --- |
| Wang 2015 | BCC | PDT | Surgery, cryotherapy, topical therapy, placebo | Complete clearance | 7 | 667/833 | 0.770 (0.524, 1.133) | 0.185 | 0.533 | 0.008 | ns | CL | 0.770 (0.524, 1.133) | 0.185 | 0 |
| Wang 2020 | BCC | MAL-PDT | Surgery, ALA-PDT, placebo, cryotherapy, imiquimod | AEs | 5 | 421/406 | 1.467 (1.022, 2.107) | 0.038 | 0.980 | 0.055 | IV | CL | 1.039 (0.666-1.620) | 0.866^†^ | 2 |
| Yu 2023 | Unresectable extrahepatic cholangiocarcinoma | PDT with chemotherapy | PDT alone | OS | 3 | 185 | 0.363 (0.152, 0.870) | 0.038 | 0.004 | 0.024 | IV | L | 0.450 (0.315-0.642) | <0.001 | 2 |
| Yu 2023 | Unresectable extrahepatic cholangiocarcinoma | PDT with chemotherapy | PDT alone | AEs (photosensitivity reaction) | 3 | 73/114 | 1.107 (0.575, 2.129) | 0.574 | 0.003 | 0.622 | ns | L | 1.292 (0.495-3.374) | 0.601 | 2 |
| Zou 2016 | BCC | PDT | Surgery | 2-year recurrence | 3 | 189/192 | 1.946 (1.376, 2.753) | 0.014 | 0.037 | 0.917 | IV | L | 1.946 (1.376, 2.753) | 0.014 | 0 |
| Zou 2016 | BCC | PDT | Surgery | 1-year complete response | 3 | 157/159 | 0.646 (0.544, 0.767) | 0.008 | 0.047 | 0.710 | IV | L | 0.9412 (0.879-1.009) | 0.089 | 2 |
| Wang 2020 | BCC | MAL-PDT | Surgery, ALA-PDT, placebo, cryotherapy, imiquimod | 1-year complete response | 6 | 693/730 | 0.720 (0.558, 0.93) | 0.012 | 0.028 | 0.739 | IV | CL | 0.720 (0.558, 0.930) | 0.012 | 0 |
| Zhong 2020 | Bowen’s disease | PDT | 5-Fu | Lesion reduction | 4 | 176/88 | 5.266 (0.578, 47.994) | 0.097 | 0.021 | 0.003 | ns | L | 2.580 (0.497, 13.388) | 0.259 | 2 |
| Zhong 2020 | Bowen’s disease | PDT | 5-FU, cryotherapy | Lesion reduction | 6 | 320/199 | 3.193 (1.230, 8.288) | 0.026 | 0.005 | 0.006 | IV | L | 2.158 (1.465-3.178) | < 0.001 | 3 |
| Ou-yang 2023 | BCC and SCC | MAL-PDT | YAG-AFL-PDT | Response at 12 months | 3 | 57/60 | 0.378 (0.175, 0.818) | 0.032 | 0.004 | 0.005 | IV | L | 0.563 (0.316, 1.002) | 0.051^†^ | 2 |
| Gu 2021 | BCC and SCC | Laser-assisted PDT | Conventional PDT | Complete response rate | 5 | 139/128 | 2.349 (1.639, 3.368) | <0.001 | 0.895 | 0.096 | IV | L | 1.511 (0.950-2.402) | 0.081^†^ | 2 |
| Gu 2021 | SCC | Laser-assisted PDT | Conventional PDT | Complete response rate | 3 | 102/91 | 2.748 (2.187, 3.452) | 0.003 | 0.777 | 0.086 | IV | L | 1.574 (1.290, 1.919) | < 0.001 | 2 |

^†^ The statistical significance of the pooled effect size changed after trim-and-fill analysis.

^§^ The number of studies added by the trim-and-fill analysis.

^ƒ^ In the original meta-analysis, the number of included studies on specific intervention measures and outcomes.

**^£^** In the original meta-analysis, the number of patients (intervention/comparison) on specific intervention measures and outcomes.

**^‡^** The p-value resynthesized from the original meta-analysis.

**^¶^** The p-value of the egger test for the meta-analysis, which reflects publication bias or selective reporting bias.

**^€^** This is the P-value of the excess significance test for the meta-analysis, which reflects publication bias or selective reporting bias.

Abbreviation: AEs, Adverse events; ALA, 5-Aminolevulinic acid; AMSTAR 2, assessment of multiple systematic reviews; BCC, basal cell carcinoma; CI, confidence interval; CL, critical low; ES, effect size; L, low; MAL, methyl aminolevulinate; OS, overall survival; PDT, photodynamic therapy; RA, re-analyze; SCC, squamous cell carcinoma; TES, test of excess significance; TFMA, trim-and-fill method analysis; YAG-AFL, erbium: yttrium-aluminum-garnet ablative factional laser; 5-FU, 5-Fluorouracil; IV, weak evidence (class IV); ns, non-significant (class ns).
